# Contrasting adaptations of soil prokaryotes and arbuscular mycorrhizal fungi in saline wildland and non-saline farmland

**DOI:** 10.1016/j.fmre.2025.02.009

**Published:** 2025-02-25

**Authors:** Huanhuan Zhu, Kobilov Fazliddin, Qiushi Li, Cong Wang, Peilin Chen, Jianxia Yang, Qiang Dong, Xingchun Li, Davranov Kakhramon, Gulyamova Toshkhon, Bo Yu, Hua Xiang, Cheng Gao

**Affiliations:** a State Key Laboratory of Mycology, Institute of Microbiology, Chinese Academy of Sciences, Beijing 100101, China; b College of Life Sciences, University of Chinese Academy of Sciences, Beijing 100049, China; c Institute of Microbiology Academy of Science of the Republic of Uzbekistan, Tashkent 100128, Uzbekistan; d CAS Key Laboratory of Microbial Physiological and Metabolic Engineering, Chinese Academy of Sciences, Beijing 100101, China; e State Key Laboratory of Microbial Resources, Institute of Microbiology, Chinese Academy of Sciences, Beijing 100101, China

**Keywords:** Tillage, Salinity, Diversity, Co-occurrence network, Community assembly, Functional traits

## Abstract

Understanding the environmental adaptation of microbiota is essential for ecosystem management. However, the difference in community structure of prokaryotes and arbuscular mycorrhizal (AM) fungi between saline wildland and non-saline farmland remains unclear. Here, we performed 18S rDNA sequencing to detect AM fungi and 16S rDNA sequencing to detect prokaryotes for soils collected in wildlands (bareland, *Suaeda,* and *Tamarix* lands) and farmlands (cotton and maize lands) in Uzbekistan. Higher beta diversity, stronger effects of ecological drift, and a simplified co-occurrence network were detected for AM fungal community in farmland compared to wildland, likely due to the formation of patchy communities with a small number of individuals (low abundance) caused by tillage and reduced universality of AM fungal community dynamics, i.e., interactions among AM fungi and their environment, might be stable in wildland but not in farmland. In contrast, lower beta diversity, weaker effects of ecological drift and dispersal limitation, and a complexified co-occurrence network were detected for prokaryotic community in farmland than in wildland, likely due to community homogenization caused by tillage that increases the odds of encounters between different prokaryotic taxa. Besides, larger average genome size and higher community-weighted rDNA copy number were detected for prokaryotes in wildland than in farmland, alongside the rise of *Acinetobacter* members and functional enrichment of stress (salinity) tolerance. Our findings suggest that prokaryotic and AM fungal communities are actively responsive to the changes in salinity, resources, and disturbance, and this pattern could be harnessed for saline land reclamation practices.

## Graphical abstract

**Figure:**
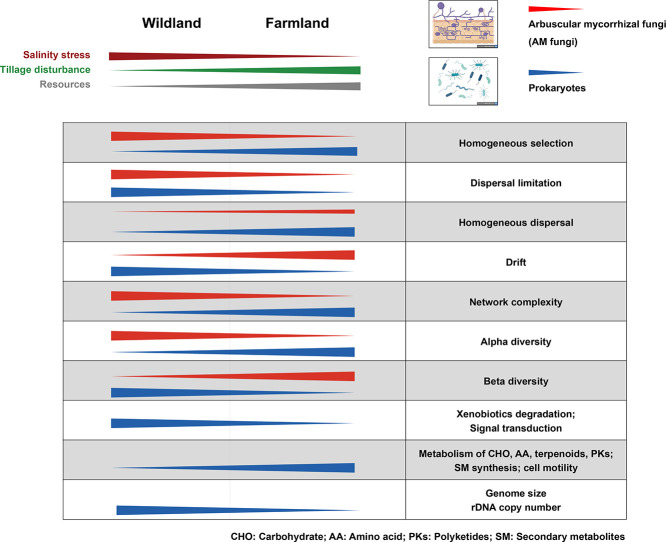

## 1 Introduction

Nearly 10% of the world population (approximately 757 million people) is suffering from hunger [1], and zero hunger is one of the major goals of the sustainable development goal (SDG) [2]. Soil salinity is a global threat to sustainable development [3], and the reclamation of saline habitats that cover up to 7% (∼1.0 × 10^9^ ha) of terrestrial land of the planet is a promising far-reaching solution for sustainable development [4]. Microbes that are widely distributed in soil and plants are shaped by the environment and, in turn, influence and reshape the environment they inhabit [[5], [6], [7], [8]]. In particular, arbuscular mycorrhizal (AM) fungi form symbiotic associations with at least 70% of plant species [9], through which they enhance plant absorption of water and nutrients [[10], [11], [12]], protect plants from biotic and abiotic stresses [[13], [14], [15]], and improve soil structure [16]. Non-saline farmland (hereafter farmland), as compared with saline wildland (hereafter wildland), typically features lower salinity stress, greater tillage disturbance, and higher resource availability [17]. Conversions between wildland and farmland involve shifts in soil salinity, nutrients, tillage, and plant coverage and identity [[18], [19], [20]], which might exert further effects on AM fungal and prokaryotic communities. Thus, unveiling the adaptations of microbiome to farmland and wildland could be conducive to saline land reclamation practices.

Alpha and beta diversities are important properties in ecology [21], and might be affected by salinity, resources, tillage, and plants. For prokaryotic communities, previous studies showed that more species could be harbored in environments with more soil resources and that tillage practice could result in community homogeneity depicted by lower beta diversity [22,23]. Thus, we speculate that alpha diversity would be higher, but beta diversity would be lower in farmland as compared to wildland, as more species can be harbored by more soil resources and species can be more homogeneously dispersed by tilling in farmland than in wildland. For AM fungal communities, a number of previous studies showed that AM fungal richness decreased with soil nutrient enrichment [24,25], and that AM fungal dispersal via soil common mycorrhizal hyphal network was reduced by tillage activity [[26], [27], [28], [29], [30]]. Besides, AM fungal richness has been found to vary with plant richness and identity [[31], [32], [33], [34]]. Thus, we speculate that alpha diversity would be lower but beta diversity would be higher in farmland as compared to wildland, as species dispersal via hypha can be reduced due to the disruption of common mycorrhizal network in farmland than in wildland.

Environmental response of microbiome might also involve biotic associations. For prokaryotic communities, previous studies showed that agricultural disturbances caused by land-use change can enhance the complexity of bacterial networks [35,36]. Thus, we speculate more biotic associations in farmland than in wildland, as soil homogenization caused by tillage would increase biotic homogenization [23], increasing the odds of encounters between prokaryotic taxa. For AM fungal communities, a previous study found that fallow land possessed a more complex AM fungi network than cropland [37]. Besides, universal community dynamics, a feature that remains fundamentally unchanged across different subjects, was previously detected for AM fungal communities in natural ecosystems but not in agricultural ecosystems [38]. Thus, we speculate fewer biotic associations in farmland than in wildland, as patchy AM fungal communities with a small number of individuals (low abundance) resulting from tillage would weaken the universality of community dynamics. To our knowledge, a systematic comparison of network complexity of prokaryotes and AM fungi between wildland and farmland remains lacking. Based on these speculations, we hypothesize higher beta diversity and lower network complexity of AM fungi due to ecological drift, but lower beta diversity and high network complexity of prokaryotes due to homogenous dispersal in farmland than in wildland.

Environmental adaptation of microbes often involves shifts in functional traits such as genome size, GC content, and ribosomal DNA (rDNA) copy number [39,40]. A number of studies have documented the associations of bacterial average genome size with soil pH, soil salinity, and the carbon: nitrogen ratio of soil [22,41,42]. In the comparison between wildland and farmland, one might expect a larger prokaryotic average genome size with versatile functions to tolerate saline stress [22] but a smaller prokaryotic average genome size with simplified functions to adapt to resource-poor conditions [41]. Thus, the difference in average genome size between wildland and farmland cannot be simply deduced due to the coupling of saline stress and low resource availability that potentially exert opposing effects on average genome size. As several studies found that high community-weighted rDNA copy number was associated with great disturbance and high resource availability [[43], [44], [45]], one might simply expect a higher community-weighted rDNA copy number in farmland than in wildland. However, to our knowledge, whether and how microbial average genome size and community-weighted rDNA copy number differ between wildland and farmland remains unresolved.

Uzbekistan, the most populated country in Central Asia that connects Europe, the Middle East, and East Asia, suffers from soil salinity caused largely by massive irrigation and inadequate drainage during the cultivation of cotton [[46], [47], [48]]. In this study, using metabarcoding sequencing to target prokaryotes and AM fungi in soils collected from wildland and farmland in Uzbekistan, we explored one question regarding functional traits and tested a hypothesis regarding beta diversity and network, i.e., higher beta diversity and lower network complexity of AM fungi due to ecological drift, but lower beta diversity and higher network complexity of prokaryotes due to homogenous dispersal in farmland than in wildland.

## 2 Materials and methods

### 2.1 Sites and soil sampling

**Fig. 1:**
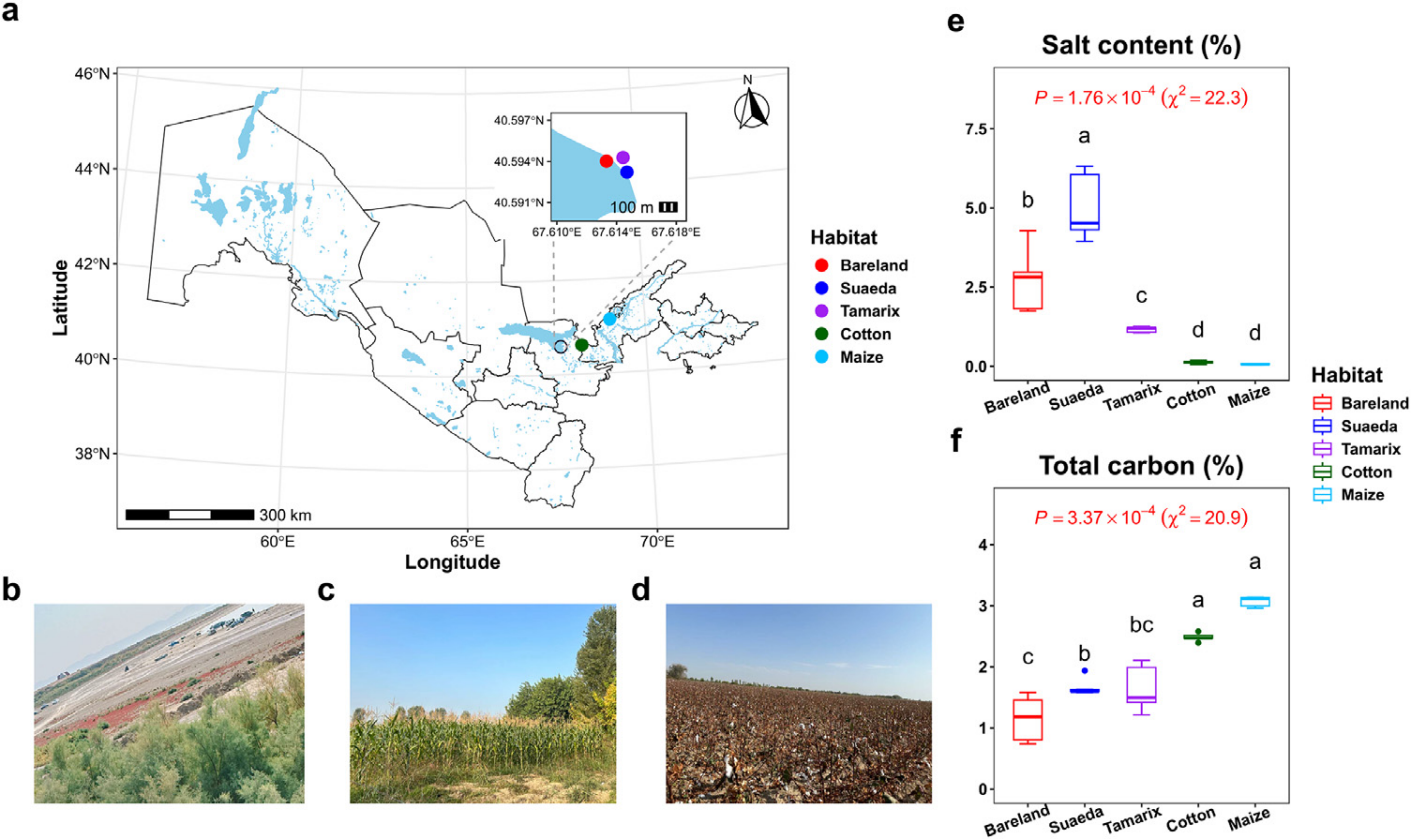
**Site, sampling, and soil properties.** a Location of two farmland sites (site #1, maize land, colored in sky blue, and site #2, cotton land, colored in green) and one wildland site (site #3, open circle). b The wildland site #3 at the eastern shore of the Ayder Lake includes three habitats, i.e., bareland (colored in red), naturally vegetated by *Suaeda* (colored in blue), or naturally vegetated by *Tamarix* (colored in purple). c-d Photos of cotton and maize lands. e Boxplot shows that soil salt content is highest in *Suaeda* land and bareland, followed by *Tamarix* land, and lowest in maize and cotton lands. Note that significant differences were detected between any two habitat pairs, except for the pair of maize and cotton lands. f Boxplot shows that total carbon is significantly higher in maize and cotton lands as compared to *Suaeda, Tamarix* lands, and bareland, whereas no significant difference was detected between maize and cotton lands, between *Suaeda* and *Tamarix* lands, and between *Tamarix* land and bareland. The differences were detected by the Kruskal-Wallis test with the *P*-value adjusted by the Bonferroni method. The map data for Uzbekistan is sourced from OpenStreetMap (https://www.openstreetmap.org) and the Humanitarian Data Exchange (https://data.humdata.org). Photo credit: Cheng Gao. (For interpretation of the references to colour in this figure legend, the reader is referred to the web version of this article.)

We collected soil samples from three sites in Uzbekistan. Farmland samples were collected from site #1 (41.14°N, 69.02°E) planted with maize, and site #2 (40.62°N, 68.20°E) planted with cotton. Wildland samples were collected from site #3 (40.59°N, 67.61°E) along a primary successional gradient from bareland to land vegetated by *Suaeda* and *Tamarix*. The farmlands were mono-dominated by maize or cotton, accompanied by weeds (Fig. 1c, d), and the wildlands were bareland or mono-dominated by *Suaeda* or *Tamarix* (Figs. 1b and S1c-e). For each of the five habitat types (maize land, cotton land, bareland, *Suaeda* land, and *Tamarix* land), five bulk soil samples with a distance > 3 m from each other were collected and sealed into plastic bags, transferred to the laboratory and stored at −20 °C.

### 2.2 Soil physicochemical properties

Soil pH was measured in a 1:5 (w/v) soil-to-water suspension using a pH meter (Sartorius PB-10, Germany). Soil salt content was measured in a 1:5 (w/v) soil-to-water suspension using the oven-drying method (Bluepard DHG-9141A, China). Total carbon (TC) and total nitrogen (TN) were measured using an elemental analyzer (Elementar vario EL, Germany). Total phosphorus (TP) was measured using the alkali fusion–Mo-Sb Anti spectrophotometric method (PerkinElmer Lambda25, USA). Available phosphorus (AP) was measured using the Sodium hydrogen carbonate solution-Mo-Sb anti-spectrophotometric method (PerkinElmer Lambda25, USA).

### 2.3 DNA extraction and sequencing

DNA of each sample was extracted using the DNeasy PowerSoil Pro Kit (MoBio, Carlsbad, CA, USA) following the manufacturer's instructions modified with a 40 s homogenization (6 m s^-1^) using the homogenizer (MP FastPrep-24 5 G) and a 10 min water bath at 70 °C. The concentration and quality of extracted DNA were determined by NanoDrop2000 (Thermo Fisher Scientific, Waltham, MA, USA). AM fungal community was targeted by nested PCR amplification with the primer pair GeoA2/AML2 [49,50] in the first round, and primer pair NS31/AMDGR [51,52] in the second round. Prokaryotic, fungal, and protistic communities were targeted by PCR amplification with the primer pairs 515F/806R [53], fITS7/ITS4-Fun [54,55], and 18S TAReuk454FWD1/V4 18S Next.Rev [56,57], respectively. Primer sequences are listed in Table S1.

PCR was conducted with the solutions and programs listed in Tables S2 and S3. Equal volumes of PCR products from each sample were pooled and purified using gel purification kit (Axygen, CA, USA), and the concentration of purified products was then measured with a Qubit 2.0 Fluorometer (Life Technologies, CA, USA). Sequencing adapters were added to purified products using Illumina TruSeq DNA PCR-Free Library Preparation Kit (Illumina) following the manufacturer's instructions, and the libraries were subsequently sequenced on the Illumina NovaSeq 6000 System at Shanghai Personal Biotechnology Co., Ltd.

### 2.4 Bioinformatics analysis

Sequences were aligned to barcodes at the 5′ end of the forward primer to assign them to each sample using the qiime cutadapt demux-paired command in QIIME 2 v2023.7 [58]. Forward and reverse reads were merged by the fastq_mergepairs command in USEARCH v11.0.667 [59]. Primer sequences were removed using cutadapt v4.4 [60]. Quality control was carried out using the fastq_filter command (-fastq_maxee 1.0-fastq_minlen 200) in USEARCH v11.0.667 [59]. High-quality sequences were subjected to de-replication and de-singleton and then clustered into operational taxonomic units (OTUs) at a 97% sequence similarity level using the cluster_otus command in USEARCH v11.0.667 [59]. OTUs were identified by a BLAST v2.15 [61] search of the most abundant sequence representing that OTU against the MaarjAM database v2019 [62] for AM fungi, the SILVA database v138.1 [[63], [64], [65]] for prokaryotes, the UNITE database v9.0 (2023.07.25) [66,67] for fungi, and the PR^2^ database v5.0.0 [68] for protists. Fungal guilds were assigned by aligning the taxonomy of fungal OTUs with the FungalTraits database [69].

### 2.5 Statistical analysis

All statistical analyses were conducted in R v4.4.0 [70] unless stated elsewhere and visualized by the ggplot2 package v3.5.1 [71]. Reads were rarefied to the minimum number of reads across all samples, and the resulting rarefied OTU tables were used for subsequent analyses. Alpha diversity was calculated using the diversity function in the vegan package v2.6–8 [72], with differences among different habitats explored using the kruskal.test function in the agricolae package v1.3–7 [73]. Beta diversity was depicted by Bray-Curtis dissimilarity between communities of the same habitat as compared to other habitats using the betadisper function in the vegan package v2.6–8 [72], with differences among different habitats explored using the kruskal.test function in the agricolae package v1.3–7 [73]. Bray-Curtis dissimilarity of AM fungal, prokaryotic, fungal, and protistic communities was ordinated respectively by principal coordinate analysis (PCoA) and jointly by multiple co-inertia analysis (MCoIA) using the cmdscale function in the stats package v 4.4.0 [70] and the mcia function in the omicade4 package v1.42.0 [74]. Redundancy analysis (RDA) followed by envfit was performed to explore the associations between community composition and physiochemical properties of soil using the rda and envfit functions in the vegan package v2.6–8 [72].

Having detected differences in beta diversity among habitats, we explored community assembly using the iCAMP package v1.5.12 [75]. Significant phylogenetic signals were detected under the following conditions: phylogenetic signal threshold (*d*_*s*_) from 0.1 to 0.4, and minimal required bin size (*N*_min_) = 72 for AM fungi, *N*_min_ = 32 for prokaryotes, and *N*_min_ = 96 for protists. We selected *d*_*s*_ = 0.2, as the phylogenetic signal of microbial niche preference has generally been found to be significant in various environments at this threshold [[76], [77], [78]]. Differences in community assembly between wildland and farmland were tested using the Chi-square test with the prop.test function in the stats package. v4.4.0 [70].

Co-occurrence networks were constructed separately for vegetated wildland (*Suaeda* and *Tamarix* lands) and farmland (cotton and maize lands). Pairwise Spearman correlations were calculated using the corr.test function in the psych package v2.4.3 [79] for OTUs with ≥ 5 reads and occurred in at least two samples. Correlations with |r| ≥ 0.6 and *P* < 0.05 were subjected to network construction and visualization using the igraph package v2.1.1 [80,81] and the ggraph package v2.2.1 [82]. Network topological parameters were calculated using functions in the igraph package v2.1.1 [80,81]. Specifically, connectance was calculated using the edge_density function, average degree using the degree function, average path length using the average.path.length function, clustering coefficient using the transitivity function, modules using the cluster_fast_greedy function, and network modularity using the modularity function.

Prokaryotic functions were predicted using the picrust2_pipeline.py command in the phylogenetic investigation of communities by reconstruction of unobserved states (PICRUSt2) [83], with the rarefied OTU table and 16S rDNA sequences of representative prokaryotic OTUs. The average GC content and average genome size of prokaryotes were calculated by aligning sequences of representative OTUs to the GTDB database v214.1 [84] and using the rarefied OTU table. The community-weighted rDNA copy number of prokaryotes was calculated by aligning sequences of represent OTUs to the rrnDB database v5.8 [85] and calculating with the rarefied OTU table. The average genome size, community-weighted rDNA copy number, and average GC content of prokaryotes without OTU5 were calculated using the same methods, based on the rarefied OTU table with OTU5 excluded. The Wilcoxon Signed-Rank Test was conducted using the wilcox.test function stats package v 4.4.0 [70] to compare the functions (predicted Kyoto encyclopedia of genes and genomes orthologs, KOs) and functional traits of prokaryotes between wildland (*Suaeda* and *Tamarix* lands) and farmland (cotton and maize lands). Differential analysis was performed to identify differential OTUs between wildland (*Suaeda* and *Tamarix* lands) and farmland (cotton and maize lands) using the DESeq2 package v1.42.0 [86]. Spearman's correlation between univariates of physiochemical properties and microbes, as well as the Mantel test between these variables and microbial communities, were performed using the linkET package v0.0.7.4 [87].

## 3 Results

### 3.1 Physicochemical properties of sampling sites

Habitat type significantly affects soil salt content, TC, pH, TN, TP, and AP (Figs. 1e, f and S2a-d). Soil salt content is highest in *Suaeda* land and bareland, followed by *Tamarix* land, and lowest in maize and cotton lands (Fig. 1e). Note that significant differences in soil salt content were detected between any two habitat pairs, except for the pair of maize and cotton lands (Fig. 1e). TC and TP are significantly higher in maize and cotton lands than *Suaeda* land, *Tamarix* land and bareland, whereas no significant difference was detected between maize and cotton lands, between *Suaeda* and *Tamarix* lands, or between *Tamarix* land and bareland (Figs. 1f and S2b). TN is significantly higher in cotton, maize, and *Tamarix* lands than bareland and *Suaeda* land, whereas no difference was detected among cotton, maize, and *Tamarix* lands, or between bareland and *Suaeda* land (Fig. S2c). Other soil physicochemical properties are detailed in Table S4.

### 3.2 Community composition

We detected 670 AM fungal OTUs, 1,923 prokaryotic OTUs, 2,511 fungal OTUs, and 2,282 protistic OTUs. The AM fungal community is predominantly composed of *Glomus* (61.6%), *Paraglomus* (18.5%), and *Claroideoglomus* (11.3%) (Fig. S3b). The prokaryotic community is mainly composed of *Proteobacteria* (23.6%), *Planctomycetes* (14.3%), and *Actinobacteria* (14.0%) (Fig. S3c). The fungal community is mainly composed of *Sordariomycetes* (34.4%), *Dothideomycetes* (15.5%), and *Agaricomycetes* (9.4%), (Fig. S3e). Classification of guilds shows that saprotroph fungi (40.8%) and plant pathogen (20.4%) are dominant guilds in fungal community, and certain types of saprotrophs (dung and wood saprotrophs) and plant pathogens are more abundant in farmland than in wildland (Fig. S4). The protistic community is mainly composed of *Evosea* (25.1%), *Chlorophyta* (19.4%), and *Ciliophora* (12.3%) (Fig. S3g). The differential analysis identified 32 AM fungal OTUs, 15 prokaryotic OTUs, and 20 protistic OTUs, with significant differences in relative abundance between wildland and farmland (Fig. S5).

MCoIA shows adaptations of AM fungal, prokaryotic, fungal, and protistic communities to habitats, with AM fungal and prokaryotic communities contributing most to the first two axes (Fig. S6). PCoA followed by permutational multivariate analysis of variance (PermANOVA) shows that habitat type significantly influences community composition, explaining 35.6% of the variance in AM fungi, 30.3% in prokaryotes, 24.2% in fungi, and 30.6% in protists (Fig. S7). Mantel test and RDA show significant correlations between AM fungal community composition and AP, TC, TP, and the TC: TP ratio, and between prokaryotic community composition and pH, salt content, TN, TC, TP, the TN: TP ratio, and the TC: TP ratio (Figs. S8, S9).

### 3.3 Alpha diversity

**Fig. 2:**
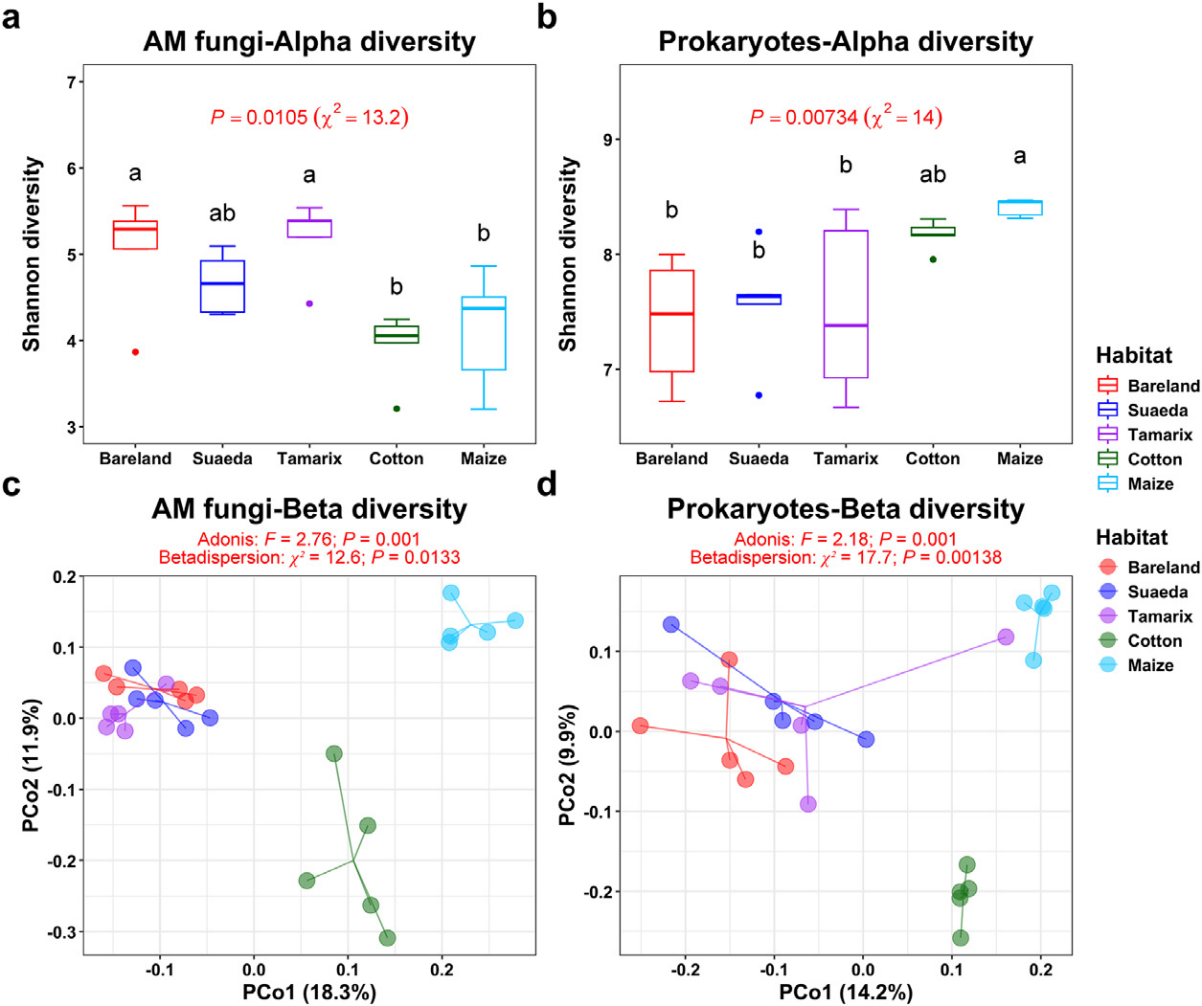
**Shrink in alpha diversity and dispersal in beta diversity of arbuscular mycorrhizal (AM) fungi in farmland and prokaryotes in wildland.** a-b Shrink in alpha diversity of AM fungi in farmland and prokaryotes in wildland. a Shannon diversity of AM fungi in cotton and maize lands is significantly lower than that in bareland and *Tamarix* land, though that in *Suaeda* land is not significantly different from any other habitats. b Shannon diversity of prokaryotes in bareland, *Suaeda,* and *Tamarix* lands is significantly lower than that in maize land, though that in cotton land is not significantly different from any other habitats. c-d Bray-Curtis dissimilarity-based principal coordinated analysis (PCoA) followed by permutational multivariate analysis of variance (PermANOVA, adonis) and beta dispersion (betadisper) show significant differences in both community composition and beta diversity for c AM fungi, and d prokaryotes between wildland and farmland. Note that the higher beta diversity of AM fungi in farmland and prokaryotes in wildland is depicted by boxplots showing the distance to centroids of replications within each condition in Fig. S9. In the beta dispersion analysis, a higher distance to the centroid reflects higher beta diversity. The differences in both Shannon diversity and beta dispersion were detected by the Kruskal-Wallis test with the *P* value adjusted by the false discovery rate (FDR) method.

One-way analysis of variance (ANOVA) shows that alpha diversities of AM fungi and prokaryotes are significantly affected by habitat type (Fig. 2a-b). Shannon diversity of AM fungi is highest in bareland and *Tamarix* land, followed by *Suaeda* land, and lowest in cotton and maize lands. No significant difference was detected among *Suaeda*, cotton, and maize lands, and among bareland, *Suaeda*, and *Tamarix* lands (Fig. 2a). Shannon diversity of prokaryotes is highest in maize land, followed by cotton land, and lowest in bareland, *Suaeda*, and *Tamarix* lands. No significant difference was detected between cotton and maize lands, and among bareland, *Suaeda, Tamarix*, and cotton lands (Fig. 2b). Consistent with the Shannon index, AM fungal richness is significantly higher in farmland (cotton and maize lands) than in wildland (bareland, *Suaeda*, and *Tamarix* lands), and fungal and protistic richness show no significant difference between farmland and wildland (Fig. S11). However, prokaryotic richness is not significantly different between farmland and wildland, though a significant difference is detected for the Shannon index (Figs. 2b and S11b). This may be because richness failed to capture the variation in evenness that significantly differed between farmland and wildland for prokaryotes (Fig. S12b). Spearman's correlation analysis shows that Shannon diversity of AM fungi significantly correlated positively with the TN: TP ratio and the TC: TP ratio, and negatively with TC and TP (Fig. S8). Shannon diversity of prokaryotes significantly correlated negatively with salt content, the TN: TP ratio, and the TC: TP ratio, and positively with TC and TP (Fig. S8).

### 3.4 Beta diversity

The betadisper test detected significant dispersion of AM fungal, prokaryotic, and protistic communities, but not of fungal community within sampling sites (Figs. 2c, d and S13). Bray-Curtis dissimilarity of AM fungal community is significantly higher in cotton and maize lands than in bareland, *Suaeda*, and *Tamarix* lands, and no significant difference is detected between cotton and maize lands, and among bareland, *Suaeda* and *Tamarix* lands (Fig. S13c). Bray-Curtis dissimilarity of prokaryotic community is higher in Bareland, *Suaeda*, and *Tamarix* lands than in cotton and maize lands, and no significant difference is detected among bareland, *Suaeda,* and *Tamarix* lands, and between cotton and maize lands (Fig. S13d). Bray-Curtis dissimilarity of protistic community exhibits a decreasing gradient with the order of *Suaeda* land, bareland, *Tamarix* land, maize land, and cotton land, and significant differences are detected between any two habitat pairs, except for the pair of *Suaeda* land and bareland (Fig. S13f).

### 3.5 Community assembly

**Fig. 3:**
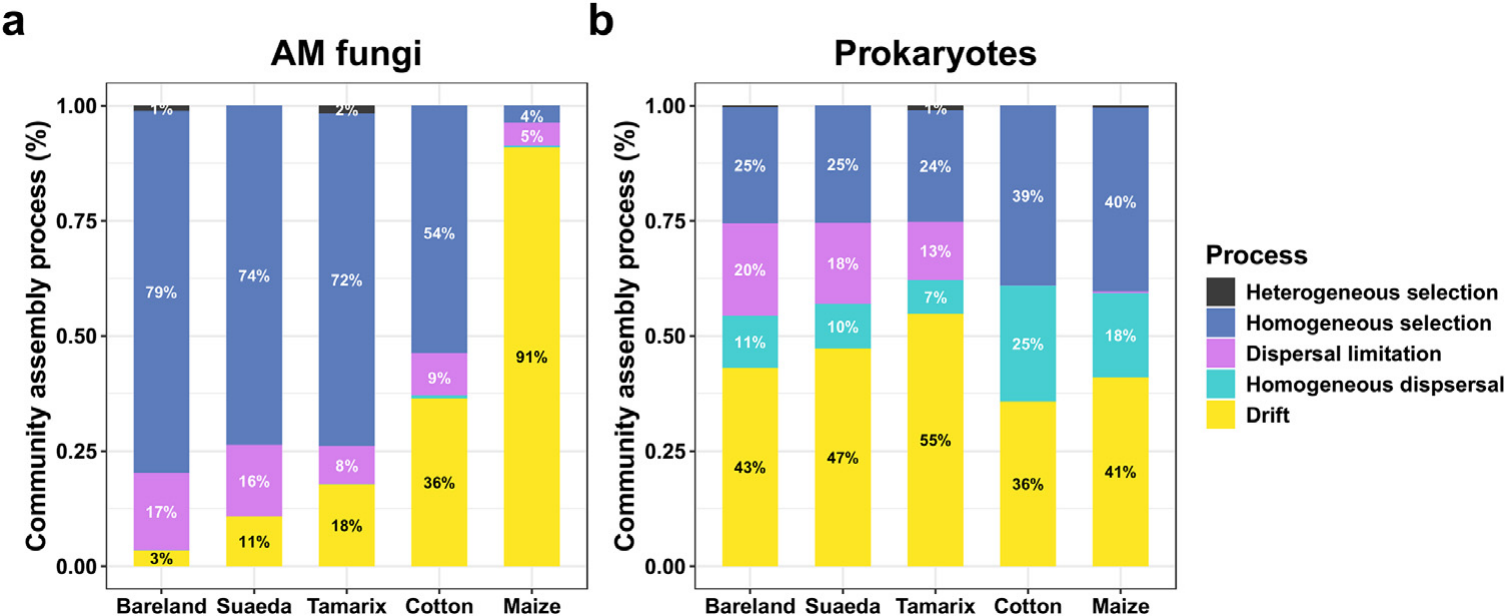
**Assembly processes of AM fungal and prokaryotic communities in wildland and farmland as detected by phylogenetic bin-based null model analysis (iCAMP).** a The relative importance of drift increases whereas that of homogenous selection and dispersal limitation decreases along the gradient of bareland, *Suaeda* land, *Tamarix* land, cotton land, and maize land. b The relative importance of homogenous selection and homogenous dispersal are higher, whereas that of dispersal limitation and drift are lower in the group of cotton and maize lands, as compared to another group formed by bareland, *Suaeda,* and *Tamarix* lands. Percentages for processes with relative importance greater than 1% are displayed in each habitat. The Chi-square test was used to test the differences in community assembly processes between wildland and farmland, as detailed in Fig. S17. (For interpretation of the references to colour in this figure legend, the reader is referred to the web version of this article).

The phylogenetic bin-based null model analysis (iCAMP) for AM fungi community shows that the relative importance of drift increases, whereas that of homogenous selection and dispersal limitation decreases along the gradient of bareland, *Suaeda* land, *Tamarix* land, cotton land, and maize land (Figs. 3a and S17a). In contrast, the iCAMP for prokaryotic community shows that the relative importance of homogenous selection and homogenous dispersal is higher, whereas that of dispersal limitation and drift is lower in the group of cotton and maize lands, as compared to another group formed by bareland, *Suaeda* and *Tamarix* lands (Figs. 3b and S17b). For protists, the iCAMP shows the relative importance of dispersal limitation is higher in bareland, *Suaeda,* and *Tamarix* lands than in cotton and maize lands, whereas the relative importance of homogeneous selection and drift is higher in cotton and maize lands than in bareland, *Suaeda,* and *Tamarix* lands (Figs. S15, S17c).

### 3.6 Co-occurrence network

**Fig. 4:**
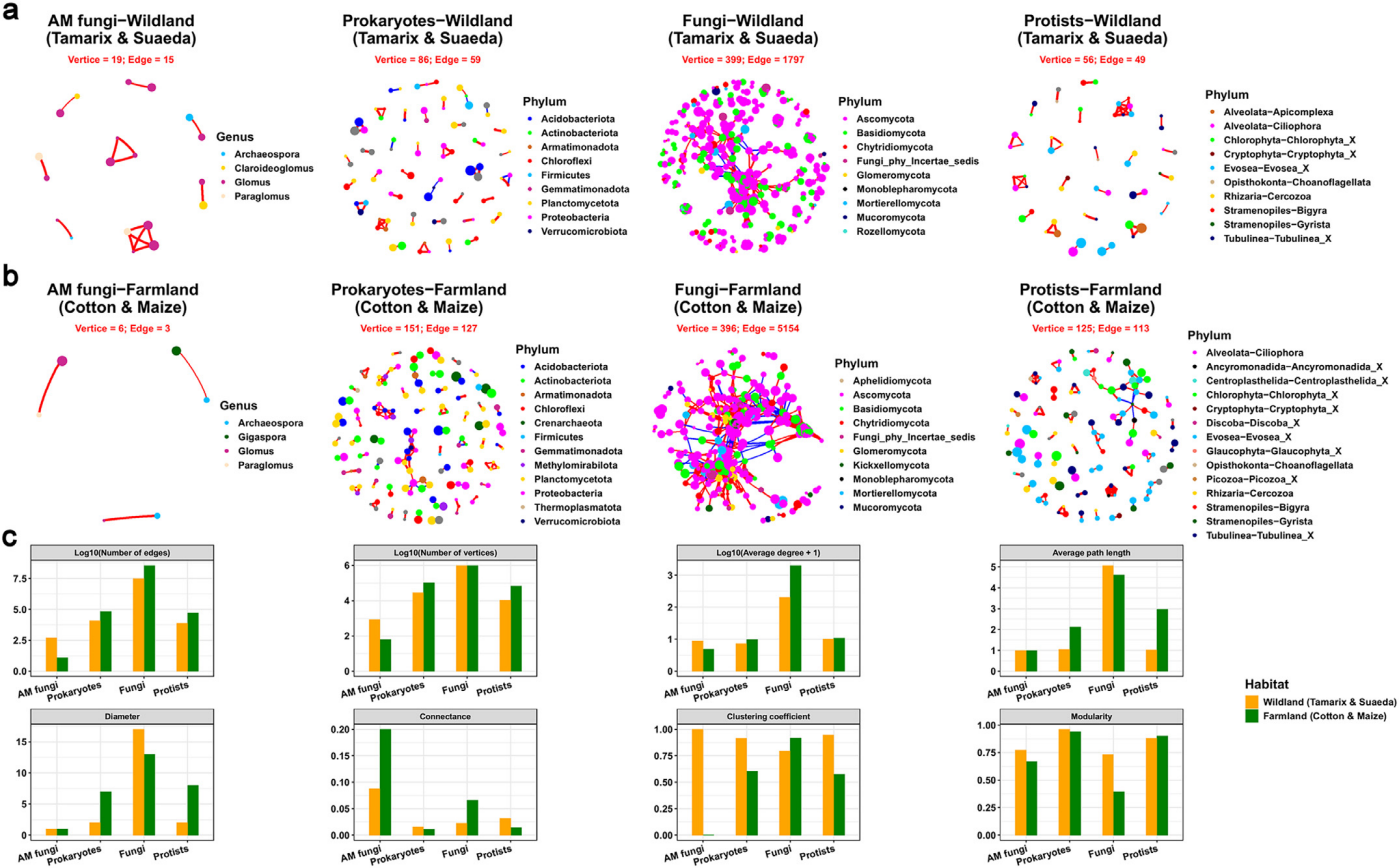
**Co-occurrence networks of all microbial communities in wildland and farmland.** Co-occurrence networks of AM fungal, prokaryotic, fungal, and protistic communities in a wildland and b farmland. The edges are colored red to inform positive correlation or blue to inform negative correlation. c Topological parameters of networks. AM fungal network shows more vertices and edges, and a higher average degree in wildland than in farmland; while the prokaryotic network, fungal network, and protistic network exhibited more vertices and edges, and a higher average degree in farmland than in wildland. The nodes of each network are colored according to prokaryotic phylum or AM fungal genus affiliation and sized according to their relative abundance. The exact values of the topological parameters are provided in Table S5. (For interpretation of the references to colour in this figure legend, the reader is referred to the web version of this article).

The co-occurrence networks in wildland are more complex for AM fungi but simpler for prokaryotes, fungi, and protists than those in farmland, as depicted by vertices, edges, and average degree (Fig. 4). There are more positive edges of AM fungi and fewer positive and negative edges of prokaryotes, fungi, and protists in wildland as compared to those in farmland (Fig. S18a, b). Further comparison of the proportion of positive edges among all edges shows that AM fungi have the same percentage of positive edges in both wildland and farmland, while prokaryotes, fungi, and protists exhibit a higher percentage of positive edges in wildland than in farmland (Fig. S18). The density plot of all Spearman's Rho shows that positive associations of AM fungi are more frequent in farmland than in wildland, whereas positive associations of prokaryotes, fungi, and protists are more frequent in wildland than in farmland (Fig. S19).

### 3.7 Putative functions

**Fig. 5:**
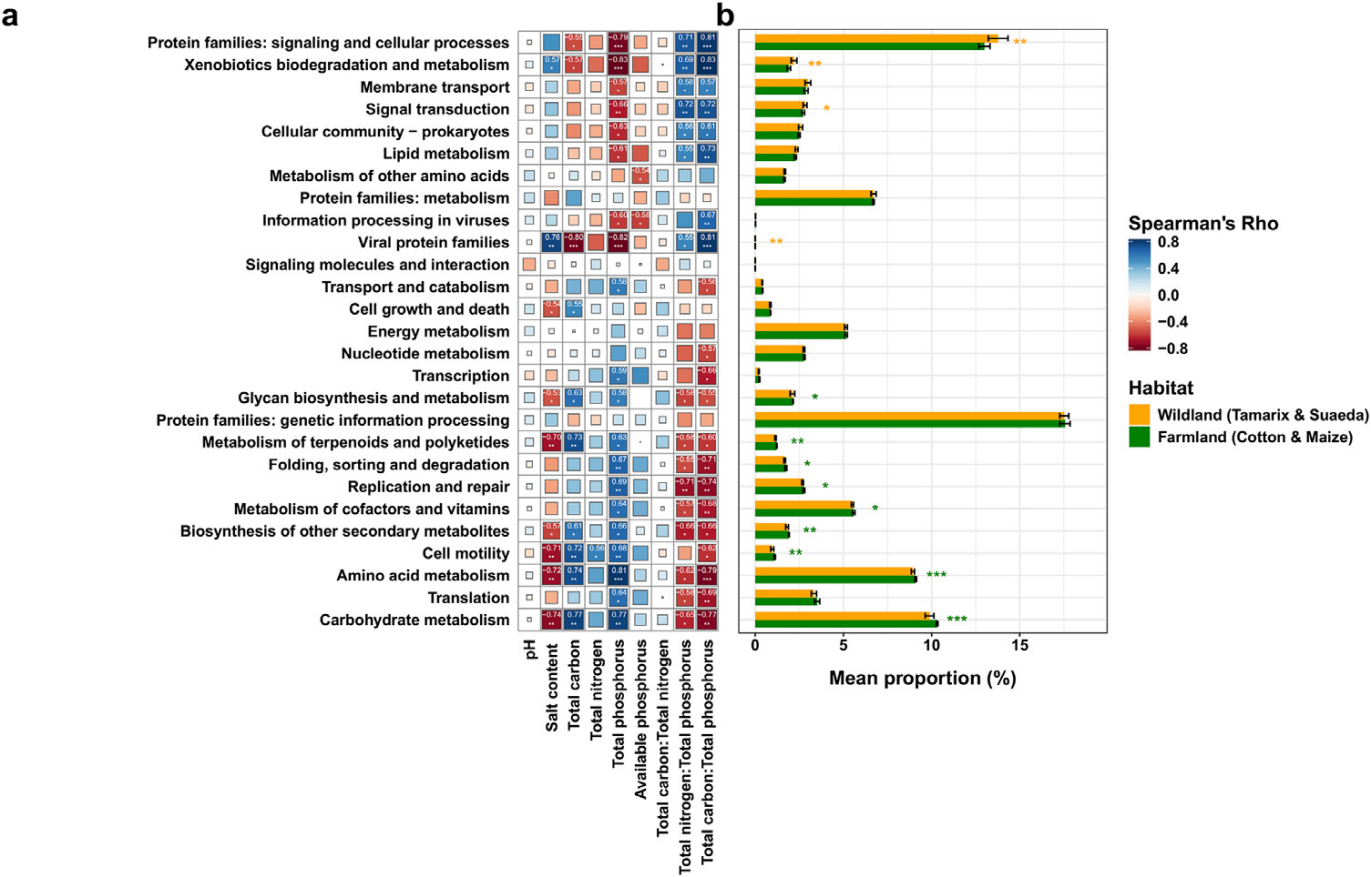
**Prokaryotes enrich xenobiotics degradation and signal transduction functions in wildland and growth and motility functions in farmland.** a Heatmap shows correlations between Kyoto Encyclopedia of Genes and Genomes Orthologies (KOs) with soil properties. b Wildland (*Suaeda* and *Tamarix* lands) enriches stress tolerance function leading by xenobiotics degradation and metabolism and signal transduction; farmland (cotton and maize lands) enriches functions involving fast growth leading by replication, cell motility, and metabolism of carbohydrate, amino acid, vitamin, cofactors, terpenoids, and polyketides. Prokaryotic functions were estimated by matching molecular taxa with the Phylogenetic Investigation of Communities by Reconstruction of Unobserved States (PICRUSt2) database. The barplot displays the differences in the relative abundance of prokaryotic functions in farmland and wildland, with the significance (false discovery rate (FDR) corrected *P*) labeled by asterisks. *P < 0.05; **P < 0.01; ***P < 0.001.

PCoA followed by PermANOVA shows that the composition of prokaryotic KOs predicted by PICRUSt2 based on 16S rDNA sequences (excluding AM fungi, fungi, and protists datasets) is significantly affected by habitat type (Fig. S20). Furthermore, we detected many significant associations between prokaryotic KOs and physicochemical properties of soil. For examples, the proportion of prokaryotic KOs involved in the functions of metabolisms of carbohydrate, amino acid, and terpenoids and polyketides, synthesis of secondary metabolites, and cell motility is significantly higher in farmland than in wildland, and significantly correlated positively with TC and TP, and negatively with salt content, the TN: TP ratio, and the TC: TP ratio (Fig. 5). The proportion of prokaryotic KOs involved in the functions of signaling and cellular processes, xenobiotics biodegradation and metabolism is significantly higher in wildland than in farmland, and significantly correlated positively with the TN: TP ratio and the TC: TP ratio, and negatively with TC and TP (Fig. 5).

### 3.8 Functional traits

**Fig. 6:**
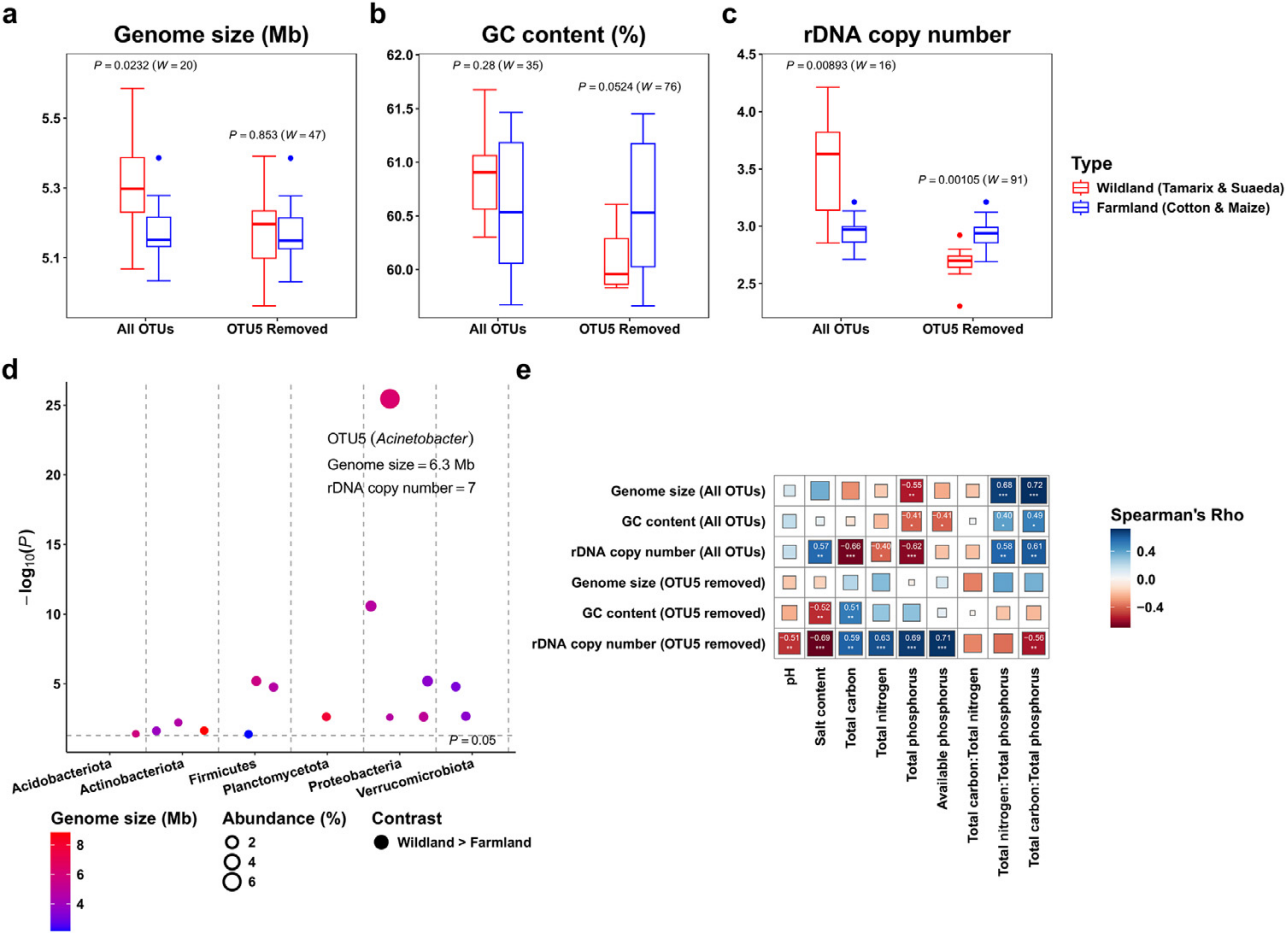
**Prokaryotic essential functional traits in wildland and farmland.** a Prokaryotic average genome size is significantly higher in wildland than in farmland across all taxa, but no significant difference is detected when the OTU5 (*Acinetobacter*) is excluded. b No significant difference in average GC content between wildland and farmland was detected for all OTUs and all OTUs but removing OTU5 (*Acinetobacter*). c Prokaryotic community-weighted rDNA copy number is significantly higher in wildland than in farmland when all taxa are considered, but it is significantly lower in wildland than in farmland when the OTU5 (*Acinetobacter*) is removed. d Manhattan plot displays 15 prokaryotic OTUs with significantly higher relative abundance in wildland than in farmland, among which OTU5 (*Acinetobacter*) features the highest -log10(*P*) value and relative abundance, and higher than median values of genome size and rDNA copy number. Note that we detected no prokaryotic OTU showing significantly higher relative abundance in farmland than in wildland. The distribution density of genome size and rDNA copy number of prokaryotic OTUs are presented in Fig. S23. e Heatmap shows correlations between soil properties and average genome size, community-weighted rDNA copy number, and average GC content of prokaryotic communities with or without OTU5. The significant correlations of average genome size with total phosphorus, the nitrogen: phosphorus ratio, and the carbon: phosphorus ratio disappeared when OTU5 was excluded. The OTU5 removal switches the correlations of rDNA copy number with salt content, the nitrogen: phosphorus ratio, and the carbon: phosphorus ratio from positive to negative, and that with total carbon, total nitrogen, total phosphorus, and available phosphorus from negative to positive. (For interpretation of the references to colour in this figure legend, the reader is referred to the web version of this article).

The average genome size and community-weighted rDNA copy number of prokaryotes are significantly higher in wildland than in farmland, and no significant difference in average GC content is detected between wildland and farmland (Fig. 6a-c). Spearman's correlation shows that average genome size is significantly correlated positively with the TN: TP ratio and the TC: TP ratio, and negatively with TP. The average GC content is significantly correlated positively with the TN: TP ratio and the TC: TP ratio, and negatively with TP and AP. The community-weighted rDNA copy number is significantly correlated positively with salt content, the TN: TP ratio, and the TC: TP ratio, and negatively with TC, TN, and TP (Fig. 6e). The contribution of *Acinetobacter* [OTU5], which exhibits significantly higher relative abundance in wildland than in farmland (Fig. 6d), to the functional traits of the prokaryotic community is assessed by calculating functional traits after excluding OTU5. The results show that no significant difference in average genome size is detected between wildland and farmland, whereas community-weighted rDNA copy number is significantly higher in farmland than in wildland when OTU5 is excluded (Fig. 6a, c).

## 4 Discussion

We found that both the alpha and beta diversities of prokaryotic and AM fungal communities exhibited opposing trends in diversity and network complexity between the wildland and farmland. These differences might be attributed to the different adaptations to the variations in disturbance, resources, and stress between the wildland and farmland of prokaryotes and AM fungi, two important microbial groups that feature dramatic differences in size, lifespan, life history, trophic mode, resource requirement, preferred niche, and reproduction process [27,88].

The difference in alpha and beta diversities of prokaryotic and AM fungal communities might be related to the difference in salinity stress and soil resources between the wildland and farmland. The alpha diversity of prokaryotes shows significant positive correlations with soil resources but a negative correlation with soil salinity (Fig. S7). Resource-rich soils likely support a greater number of species [22], whereas salt accumulation increases extracellular osmolarity, inhibiting the growth of certain microbial taxa and potentially leading to their inactivation or death [89]. In contrast, the alpha diversity of AM fungi is significantly correlated negatively with soil resources, particularly phosphorus (Fig. S7). This pattern may be attributed to the reduction in the carbon allocation from plants to AM fungi, as elevated soil phosphorus availability reduced plants' dependence on mycorrhizal pathway in phosphorus acquisition [90].

Besides, previous studies found correlations between AM fungal alpha diversity and plant diversity and identity [[31], [32], [33], [34]]. We speculate that the higher AM fungal alpha diversity in the wildland than in the farmland is unlikely associated with the even lower plant richness in the wildland (bareland or mono-dominated by *Suaeda* or *Tamarix*) than in the farmland (mono-dominated by maize or cotton, accompanied by weeds) (Fig. S11a). Previous studies showed that AM colonization on *Suaeda* is < 10% [[91], [92], [93]], whereas *Tamarix*, maize, and cotton are typically thought to be highly colonized by AM fungi [[94], [95], [96], [97]]. However, AM root colonization is not examined in our study, thus, how this difference in AM colonization of different plant species might influence our findings of diversity, network, and community assembly remains unresolved.

The higher salinity and lower resource availability in wildland as compared to farmland might act as homogenous selective forces for microbial community [98,99], which would result in lower alpha and beta diversities. This speculation of stronger homogenous selection in wildland than in farmland found strong support for AM fungal community and at least partial support for prokaryotic communities by the iCAMP-based community assembly analysis (Figs. 3a, b and S17a, b). Note that homogenous selection explains well the lower prokaryotic alpha diversity and lower AM fungal beta diversities in the wildland than in the farmland, but it fails to explain the higher AM fungal alpha diversity and lower prokaryotic beta diversities, conditions that can be well explained by tillage as detailed below.

Tillage activity in farmland can be viewed as a homogenous dispersal process for microbiota, which would result in an increase in alpha diversity and a decrease in beta diversity of soil prokaryotic community [[100], [101], [102]]. This speculation of stronger homogenous dispersal in tilled farmland than in non-tilled wildland is evidenced by our analysis of the community assembly process as determined by the iCAMP method (Figs. 3b and S17b). In line with our finding, West et al. [23] found that tillage reduced the beta diversity of soil bacterial communities by facilitating homogeneous dispersal. Note that although we found that prokaryotic alpha diversity was higher in the tilled farmland than in the non-tilled wildland, different previous studies have found that tillage can have positive, negative, or neutral effects on prokaryotic alpha diversity [23,[103], [104], [105]]. We speculate that tillage activity causes not only homogenous dispersal that promotes alpha diversity but also reduction in niche differentiation that reduces alpha diversity, and the different results of different studies might be attributed to the difference in the relative importance of these two aspects.

Over an interconnected local habitat, AM fungi are supposed to be mainly spread via hyphal extension, whereas the spore liberated to the air are supposed to be important for long-distance dispersal [30]. Tillage activity might disrupt their hyphal networks, resulting in many unconnected patches of AM fungal communities with a small number of individuals, which are likely prone to stochastic drift. The concept that tillage disrupts common mycorrhizal network has been proposed by many previous studies [26,[106], [107], [108], [109]]. The idea that communities with a small number of individuals (low abundance) are more prone to stochastic drift has been long established and has recently been validated for leaf fungal community [110]. Our speculation of stronger drift in tilled farmland than in non-tilled wildland is evidenced by our analysis of the community assembly process as determined by the iCAMP method (Figs. 3a and S17a). This stochastic drift would result in higher beta diversity and lower alpha diversity due to the random extinction of rare taxa [111]. A number of conceptual and few experiments also found the association of higher stochastic drift with higher beta diversity [110,[112], [113], [114], [115], [116], [117], [118], [119]] and lower alpha diversity [111]. In line with our finding, Peng et al. found that tillage reduced the alpha diversity of AM fungal community [120].

In contrast, our analysis found a higher stochastic drift for prokaryotic community in the wildland than in the farmland (Figs. 3b and S17b). This drift might be related to the prokaryotic communities with a low number of individuals that are limited by the low soil resource availability in the wildland. Thus, the higher beta diversity of prokaryotic community in the wildland than in the farmland might be related to both the greater homogenous dispersal in the farmland and the greater drift in the wildland.

We speculate that the different variations in network complexity of prokaryotes and AM fungi might be attributed to tilling activity. As aforementioned, the homogenous dispersal of prokaryotes in tilled farmland would increase the odds of encounters between different prokaryote members. As a result, more interactions among prokaryotic members are expected and can be depicted by a higher complexity of co-occurrence network. In contrast, as aforementioned, stochastic drift governed the assembly of patchy-distributed AM fungal community due to the disruption of soil hyphal network by tillage in farmland. We speculate that the stochastic drift of AM fungi would reduce the universality in community dynamics. In line with this speculation, Verbruggen et al. [38] found that the universality of AM fungal community was stronger in wildland than in farmland, and Gao et al. [110] found that leaf fungal community governed by stochastic drift exhibited a weaker universality as compared to that governed by deterministic processes. With a weaker universality, the outcome of biotic interaction would be unique to individual communities rather than consistent across different communities [121], a condition that can be depicted by a simplified network.

We found that prokaryotic average genome size and community-weighted rDNA copy number were significantly higher in the wildland than in the farmland (Fig. 6a, c). The larger genomes in wildland might involve versatile functions in stress tolerance and resource scavenging in resource-poor, saline environments [43]. In line with this speculation, we found that prokaryotic community in the wildland enriched gene functions of signal transduction, xenobiotics biodegradation and metabolism. In line with our findings, these gene functions are also found to be enriched for prokaryotic community in natural coastal wetlands [122]. Although prokaryotic average genome size and gene functions remain largely unclear in saline environments, our finding is consistent with several recent studies that found larger genome and more function of stress tolerance and resource scavenging in acidic, resource-poor environments [22,42,[123], [124], [125], [126]]. In contrast, prokaryotes with small genome in the farmland enriched functions involving primary and secondary metabolisms, indicating a more active lifestyle in benign, fertile environments. Similarly, Wang et al. [22] found small genome bacteria enriched gene function of energy and amino acid metabolism in resource-rich, neutral pH environment. High community-weighted rDNA copy number is considered to be related to high growth rate and is prevalent in copiotrophic environments [43,44], however, we found a higher community-weighted rDNA copy number in the wildland than in the farmland (Fig. 6c). The higher community-weighted rDNA copy number in the wildland is primarily due to the higher relative abundance of OTU5 (*Acinetobacter* sp.) in the wildland (over 10%) and high rDNA copy number of OTU5 (7 copies, far greater than the mean value of rDNA copy number in the wildland) (Figs. 6d and S3f). Previous studies also found species of *Acinetobacter* can adapt to saline environments by accumulating compatible solutes in cytoplasm to prevent excessive loss of water [127,128].

We also detected differences in certain fungal and protistic attributes between wildland and farmland. For examples, fungi and protists exhibited more complex co-occurrence networks in the farmland than in the wildland, similar to those of prokaryotes. Alike prokaryotes, protistic beta diversity is higher in the wildland than in the farmland, and the community assembly analysis shows that the higher protistic beta diversity is likely attributed to dispersal limitation. Besides, in line with previous studies [110,129], our analysis shows that plant pathogenic fungi exhibit a higher proportion of relative abundance in the farmland than in the wildland.

Our samples were collected from Uzbekistan, whereas the sequencing was conducted in China. Although this international collaboration enables the investigation of underrepresented Central Asia regions, it is challenging for us to perform further experiments to validate the proposed framework and explore the mechanisms. We found larger average genome size and higher community-weighted rDNA copy number in the wildland than in the farmland, by matching the 16S rRNA genes with GTDB and rrnDB databases. Uncertainty in this approach remains largely unclear, though Wang et al. [22] found a consistent negative association between pH and genome size detected by both the GTDB approach and the metagenome-based approach.

## 5 Conclusion

In conclusion, our comparison of farmland and wildland revealed adaptations of prokaryotes and AM fungi that feature contrasting patterns of alpha diversity, beta diversity, co-occurrence network, homogeneous selection, and drift. These differences can be largely attributed to the different operations of tillage, which likely lead to small patchy AM fungal communities but homogenize prokaryotic communities in farmland as compared to wildland. These findings might be relevant to ecosystem functioning, especially considering the frameworks of biodiversity-ecosystem functioning (BEF), beta diversity and ecosystem functioning, and community assembly and the functioning of ecosystem (CAFE). Practically, this contrasting adaptation of AM fungi and prokaryotes could be harnessed to the saline land reclamation practices, by considering exogenous addition of AM fungal inoculants originated from natural saline environments or tillage-free agriculture to protect AM fungal hyphal network. Besides, we detected a dominant taxon (*Acinetobacter* OTU5, relative abundance = 13% in wildland), whose presence strongly affected measures of average genome size and community-weighted rDNA copy number of prokaryotic communities. Several recent studies showed that average genome size and community-weighted rDNA copy number, as the surrogates of functional versatility and growth rate, were closely related to soil pH, temperature, and resources; however, whether the average genome size and community-weighted rDNA copy number of those studies are driven by a single dominant taxon remains to be addressed.

## Ethics approval and consent to participate

No ethics approval or consent to participate was required.

## Declaration of competing interest

The authors declare that they have no conflicts of interest in this work.

## Appendix Supplementary materials

Image, application 1
