# Supplementary material for: Contrasting adaptations of soil prokaryotes and arbuscular mycorrhizal fungi in saline wildland and non-saline farmland
Source: Fundam Res. 2025 Feb 25;6(4):2308–18. doi: 10.1016/j.fmre.2025.02.009 (PMC13424717; doi:10.1016/j.fmre.2025.02.009)
Supplement: Supplementary file 1 [file mmc1.pdf]

Supplementary figures

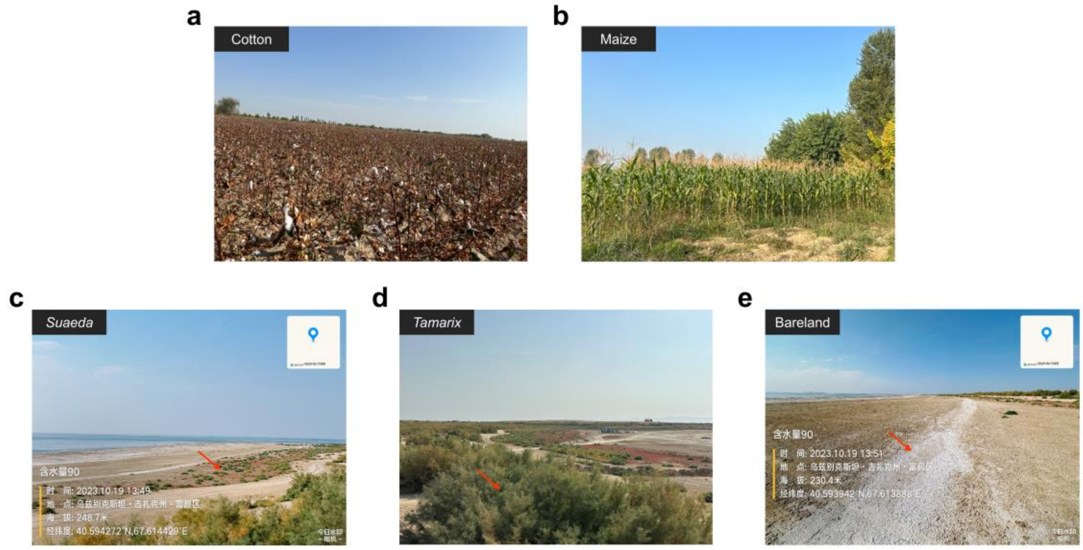

Fig. S1. Photos of sampling sites. Agricultural sites were predominantly cultivating single crops (**a** maize and **b** cotton), with only a few weeds present in the fields. Wildland sites were characterized by single plant species (**c** *Suaeda* and **d** *Tamarix*) or were barren with no vegetation (**e** bareland). Note that Fig. S1a and b are the same as that in Fig. 1b and c.

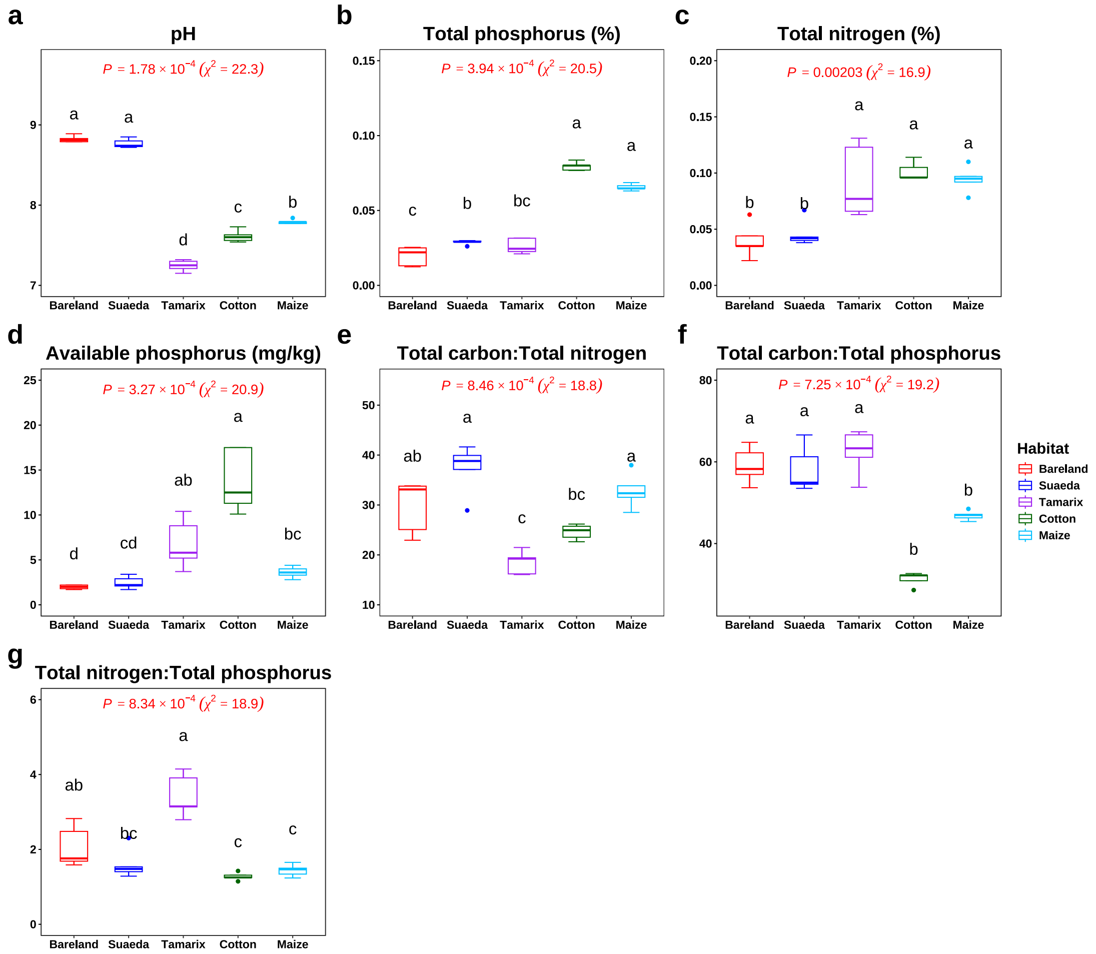

Fig. S2. Resource availability is higher in farmland than in wildland. a Soil pH is highest in bareland and *Suaeda* land, followed by maize and cotton lands, and lowest in *Tamarix* land. Note significant differences in soil pH were detected between any two habitat pairs, except for the pair of Bareland and *Suaeda* land. **b** Total phosphorus is significantly higher in cotton and maize lands than in bareland, *Suaeda* and *Tamarix* lands. **c** Total nitrogen is significantly higher in cotton, maize, and *Tamarix* lands than in bareland and *Suaeda* land. **d** Available phosphorus exhibited a decreasing gradient with the order of cotton land, *Tamarix* land, maize land, *Suaeda* land, and bareland, and significant differences were detected for any pair of non-adjacent groups. **e** The total

carbon: total nitrogen ratio exhibited a decreasing gradient with the order of *Suaeda* land, maize land, bareland, cotton land, and *Tamarix* land, and significant differences were detected for any pair of the non-adjacent groups, and no significant differences were detected between *Suaeda* and maize lands. **f** The total carbon: total phosphorus ratio is significantly lower in maize and cotton lands than in bareland, *Suaeda*, and *Tamarix* lands, but no significant difference was detected between maize and cotton lands, or among bareland, *Suaeda*, and *Tamarix* lands. **g** The total nitrogen: total phosphorus ratio exhibited a decreasing gradient with the order of *Tamarix* land, bareland, *Suaeda* land, maize land, and cotton land, and significant differences were detected for any pair of the non-adjacent groups when maize and cotton lands were treated as one group. The differences were detected by the Kruskal-Wallis test with the *P* value adjusted by the Bonferroni method.

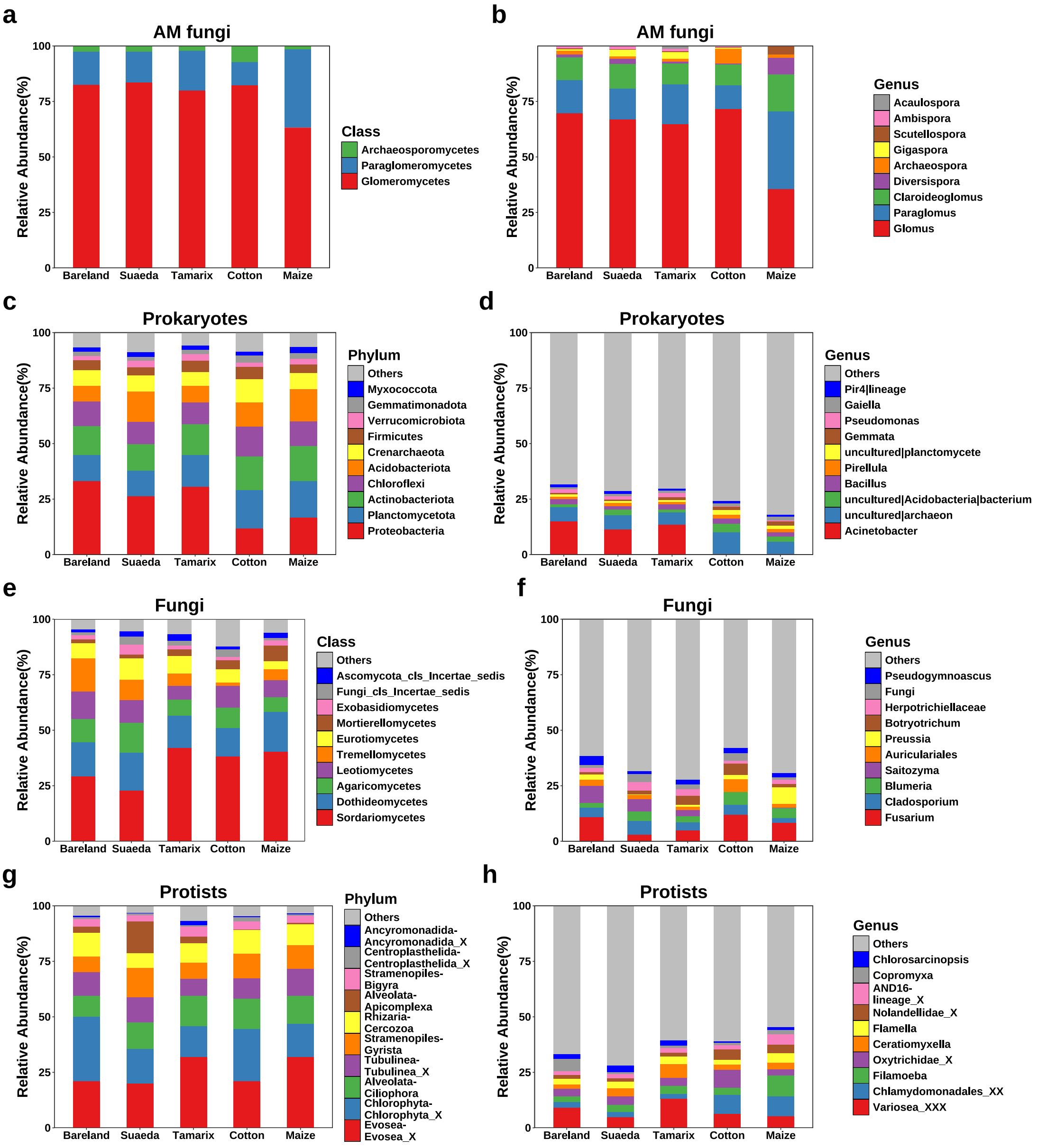

Fig. S3. Species composition of AM fungal, prokaryotic, fungal, and protistic communities. Bar plots show taxa with top ten relative abundances for **a-b** AM fungal, **c-d** prokaryotic, **e-f** fungal, and **g-h** protistic communities.

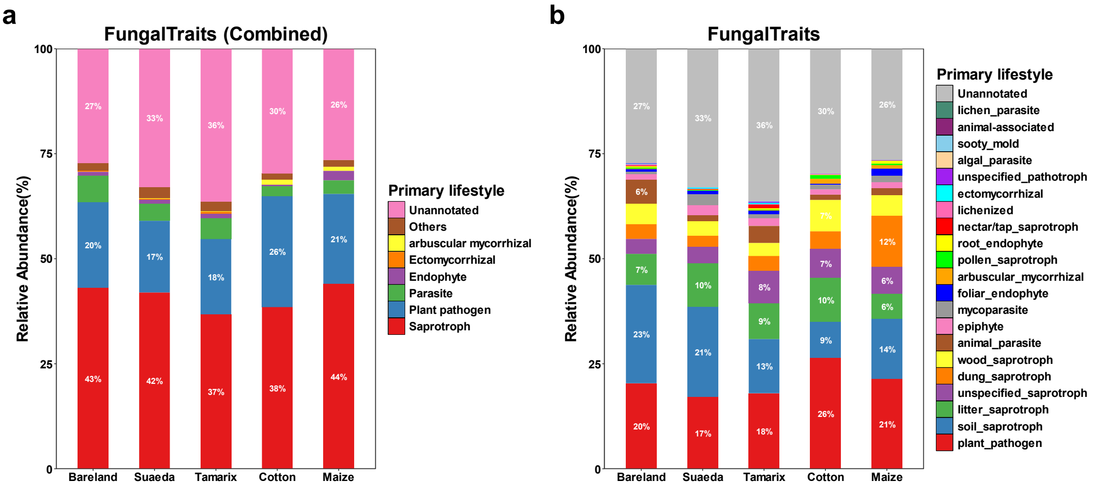

Fig. S4. Plant pathogens and certain types of saprotrophs (dung and wood saprotrophs) are more abundant in farmland than in wildland. Classification of guilds according to the FungalTraits database. **a** Guilds were combined into saprotroph, plant pathogen, parasite, ectomycorrhizal, arbuscular mycorrhizal, and others. **b** Guilds remained uncombined. Guilds with relative abundance greater than **a** 10% or **b** 5% in each habitat were added percentage text in stacked barplots.

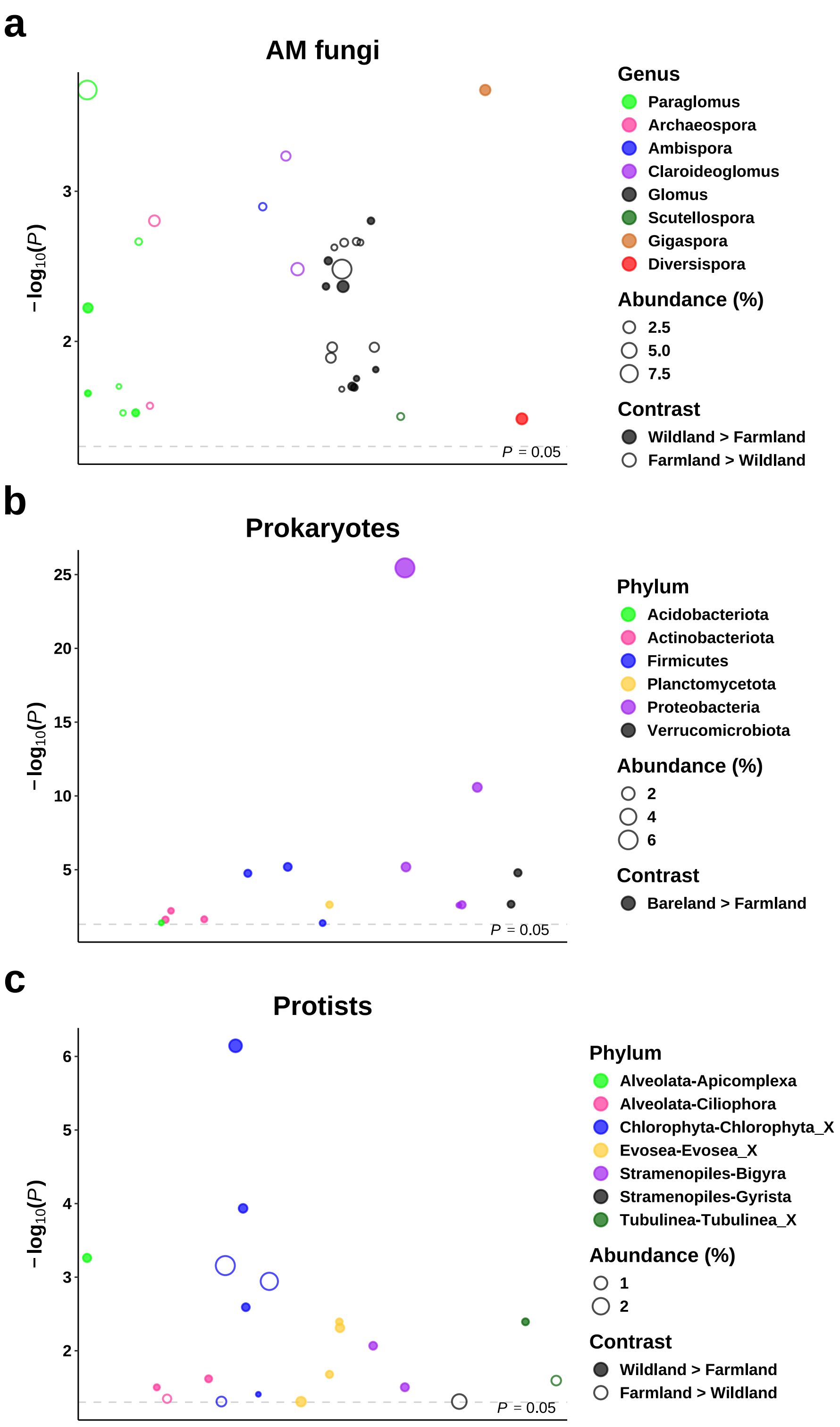

Fig. S5. Significantly differential OTUs between wildland and farmland. The Manhattan plots show OTUs with significant differences in relative abundance between wildland and farmland for **a** AM fungal community (32 OTUs), **b** prokaryotic

community (15 OTUs), and **c** protistic community (20 OTUs).

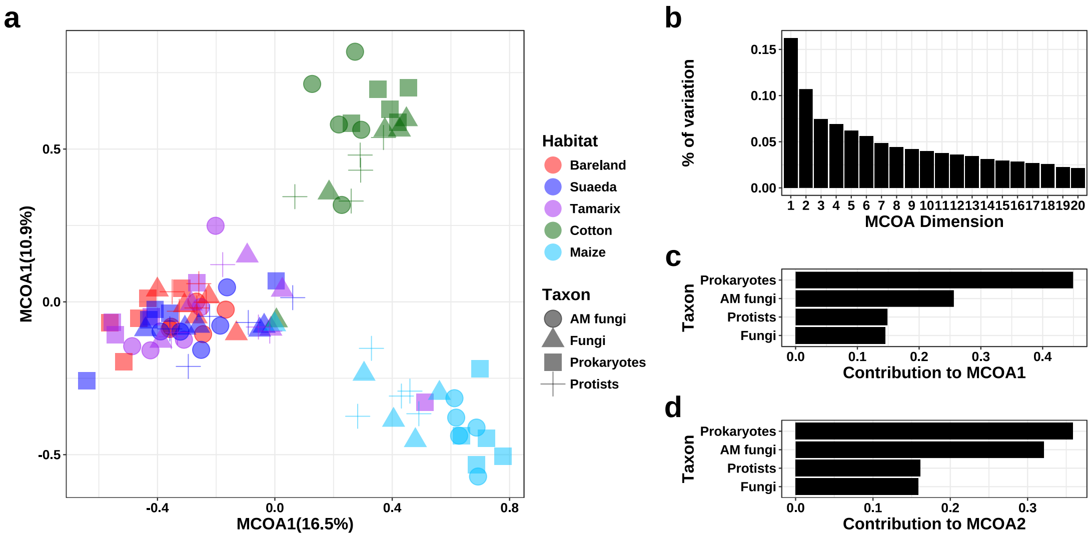

Fig. S6. Adaptions of AM fungal, prokaryotic, fungal, and protistic communities to habitats. Multiple Co-inertia Analysis (MCoIA) of all microbial communities. **a** Ordination plot of the result of MCoIA. **b** Variation captured by each MCoIA axis. **c-d** Prokaryotic and AM fungal communities exerted the highest contributions to **c** the first and **d** the second axes of MCoIA.

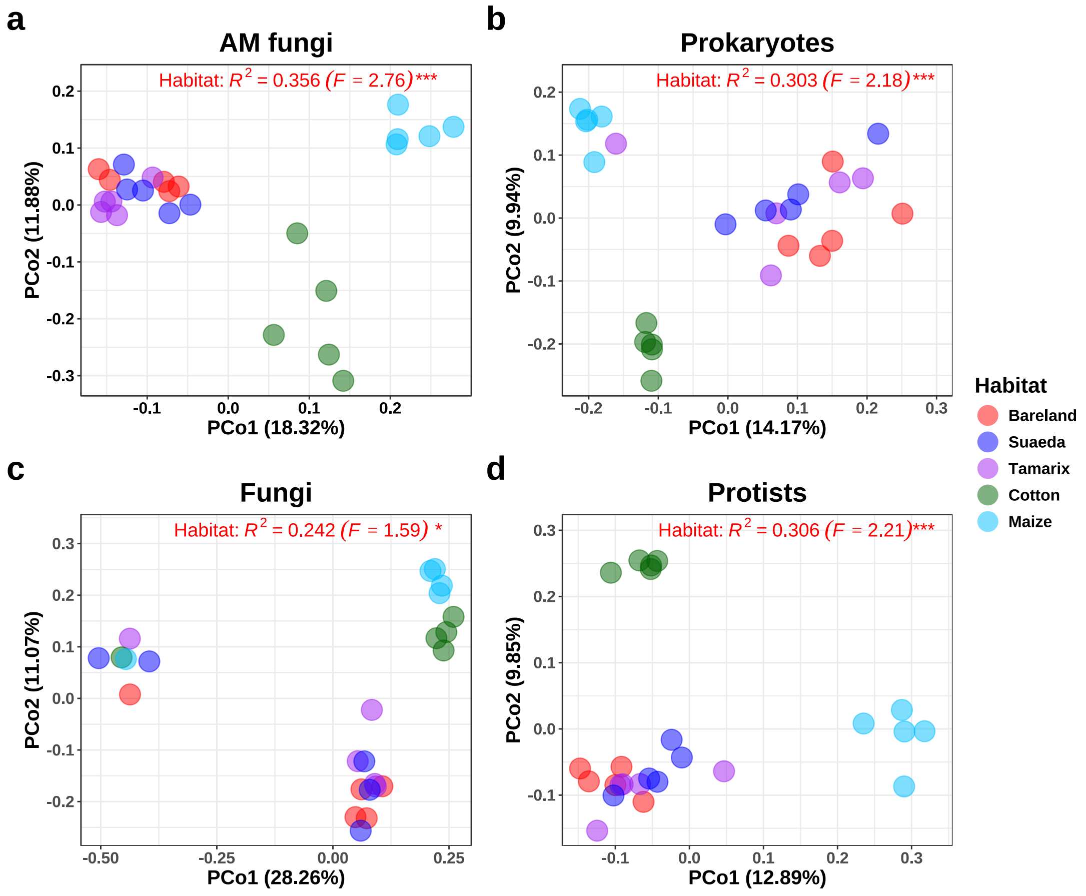

Fig. S7 Habitat significantly affects community composition of AM fungi, prokaryotes, fungi, and protists. Principal coordinates analysis (PCoA) followed by permutational multivariate analysis of variance (PermANOVA) show habitat explains over 30% variances of **a** AM fungal community, **b** prokaryotic community, **d** protistic community, and 24.2% variance of **c** fungal community.

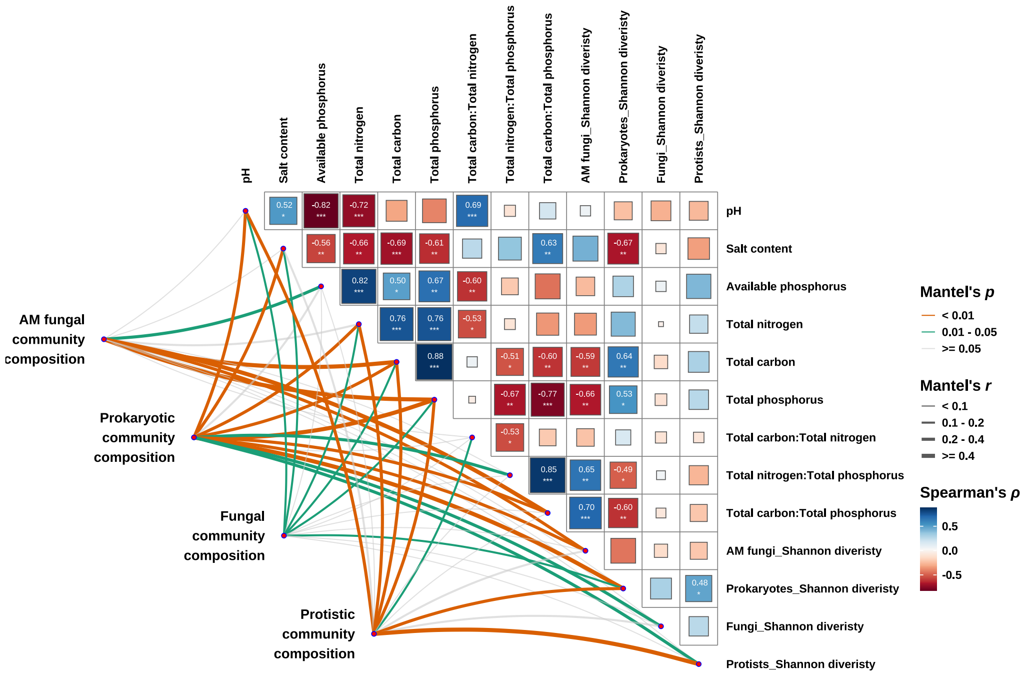

Fig. S8. Mantel test of microbial community composition and soil properties. The heatmap shows the pairwise correlations between soil properties. The lines denote the results of the Mantel test, with the line width representing Mantel's r statistic, and the color representing Speraman's correlation coefficient, the the *p* value was adjusted by the false discovery rate (FDR) method.

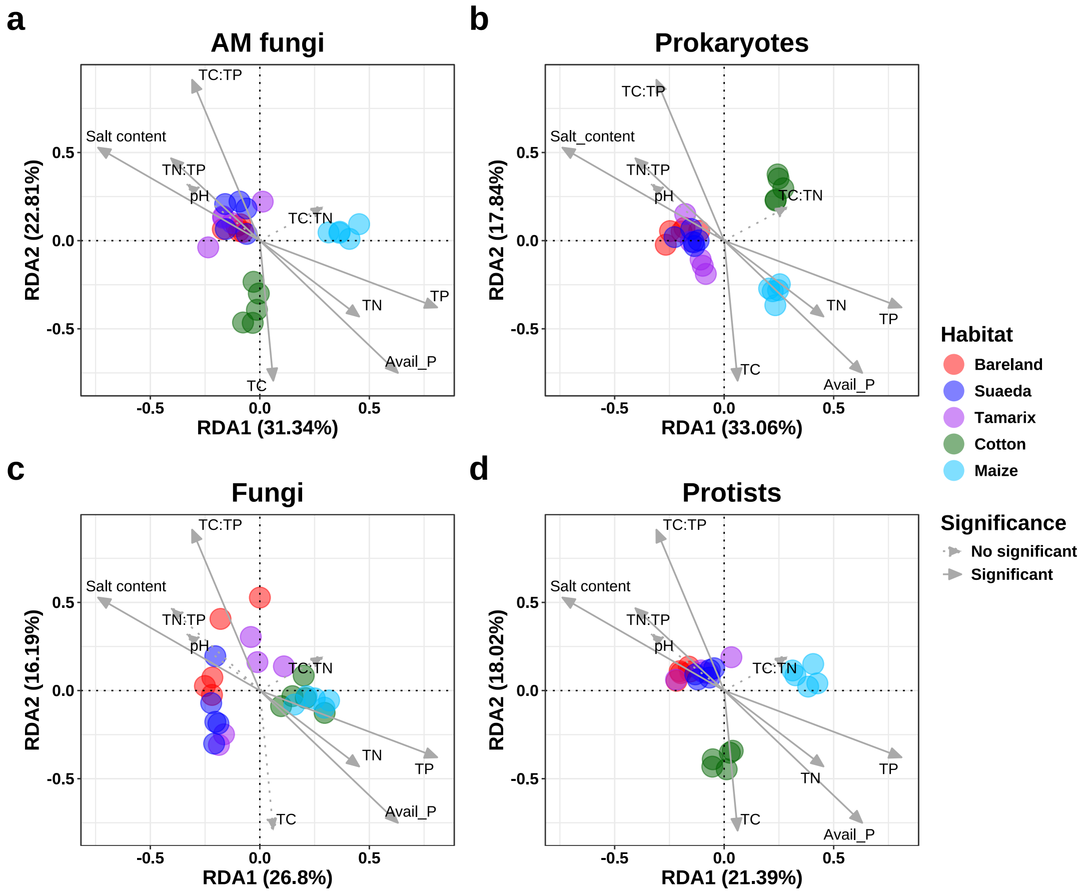

Fig. S9. Salt content, total nitrogen, total phosphorus, and available phosphorus significantly influence microbial communities. Redundancy Analysis (RDA) of **a** AM fungal community, **b** prokaryotic community, **c** fungal community, and **d** protistic and soil physiochemical properties. Significant physiochemical properties were filtered based on the results of the envfit analysis. TC: Total carbon, TN: Total nitrogen, TP: Total phosphorus, Avail_P: Available phosphorus.

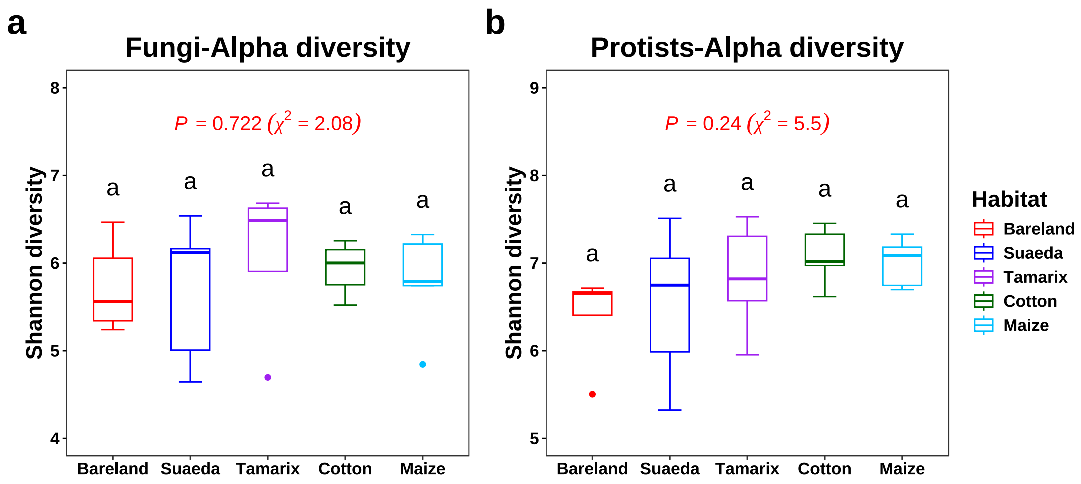

Fig. S10. No significant differences in Shannon diversity of fungi and protists were detected among five habitats. Shannon diversity of **a** fungal and **b** protistic community in five habitats. The differences were detected by the Kruskal-Wallis test with the *P* value adjusted by the false discovery rate (FDR) method.

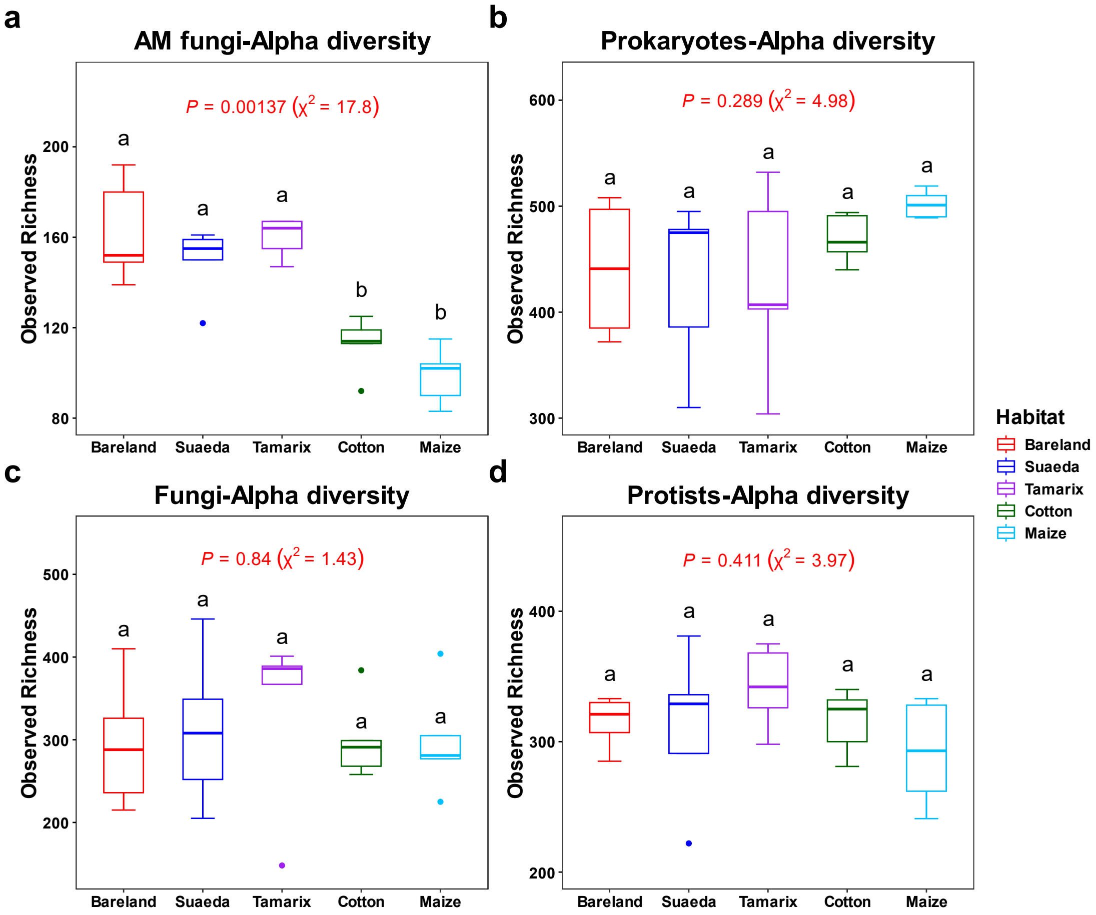

Fig. S11. Observed richness of AM fungi is significantly higher in wildland than in farmland, while no significant differences in observed richness were detected among five habitats for prokaryotes, fungi, and protists. Observed richness of **a** AM fungal, **b** prokaryotic, **c** fungi, and **d** protistic community in five habitats. The differences were detected by the Kruskal-Wallis test with the *P* value adjusted by the false discovery rate (FDR) method.

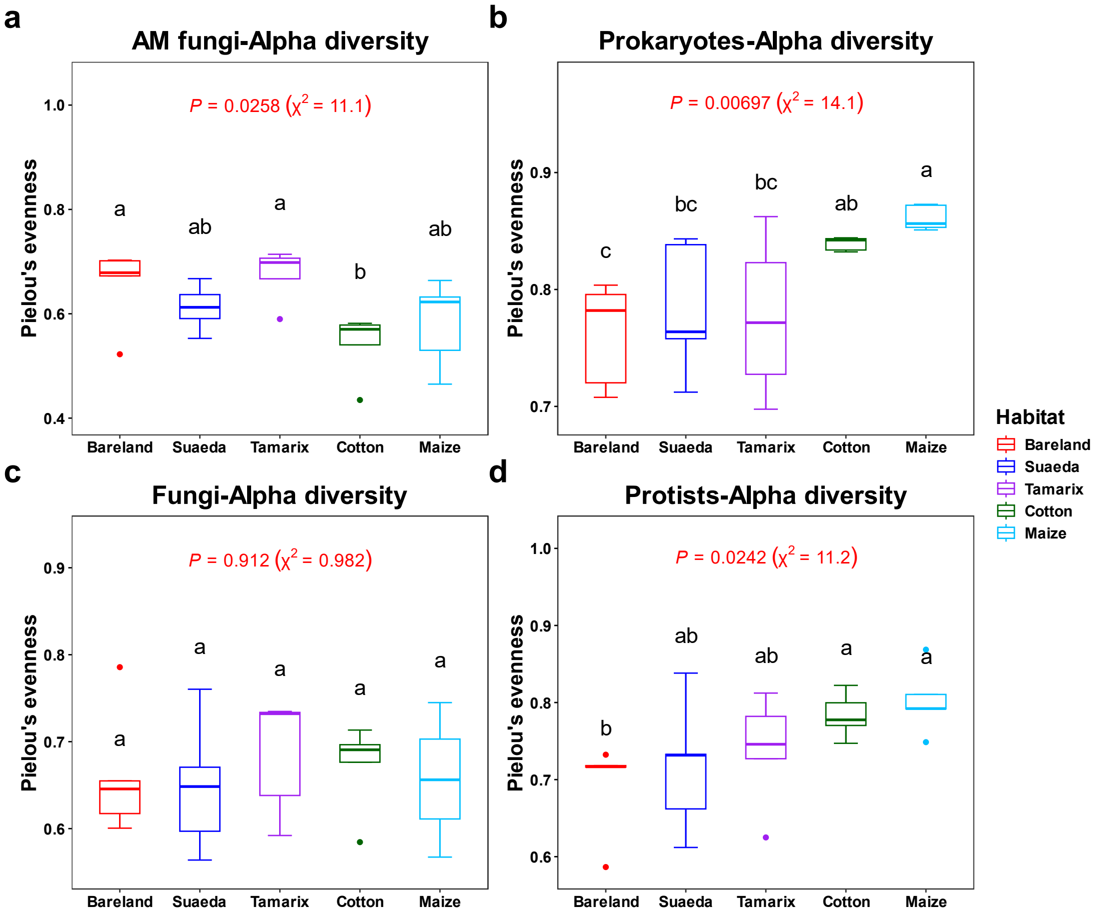

Fig. S12. Pielou's evenness of AM fungi is significantly higher in wildland than in farmland, while Pielou's evenness of prokaryotes and protists is significant higher in farmland than in wildland. Pielou's evenness of **a** AM fungal, **b** prokaryotic, **c** fungi, **d** protistic community in five habitats. The differences were detected by the Kruskal-Wallis test with the *P* value adjusted by the false discovery rate (FDR) method.

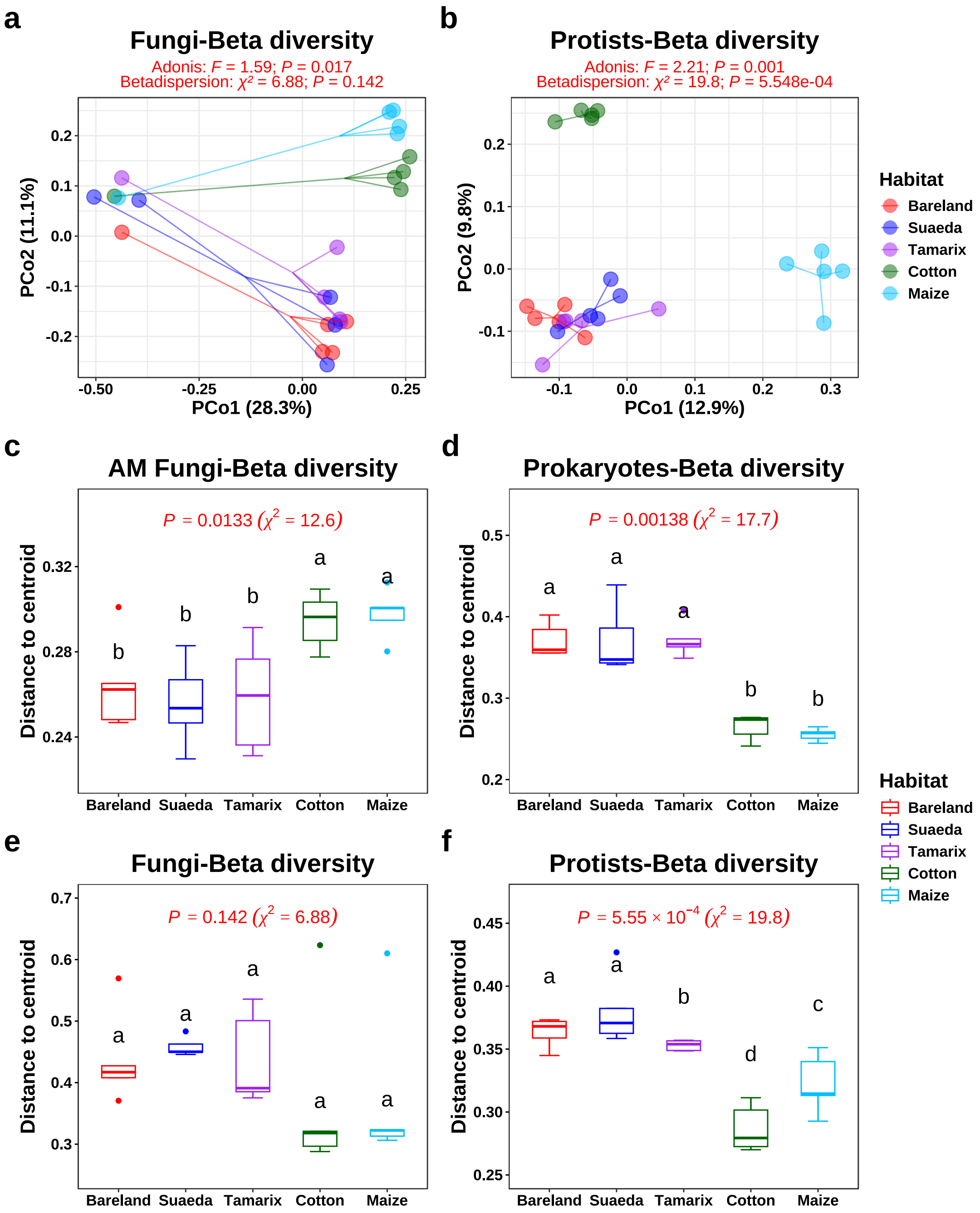

Fig. S13. Significant beta dispersion was detected among five habitats for protistic community, but not detected for fungal community. Bray-Curtis dissimilarity-based Principal Coordinated Analysis (PCoA) followed by Permutational Multivariate Analysis of Variance (PermANOVA) and beta dispersion (betadisper) show significant differences in both community composition and beta diversity for **a** fungal community

and **b** protistic community. Boxplot plots of beta dispersion show beta dispersion is significantly higher for **c** AM fungal community but significantly lower for **d** prokaryotic community in farmland than in wildland, and no significant difference in beta dispersion was detected within farmland and wildland for both AM fungal community and prokaryotic community. No significant difference in beta dispersion was detected among the five habitats for **e** fungal community. The beta dispersion of **f** protistic community decreases along the gradient of *Suaeda* land, bareland, *Tamarix* land, maize land, and cotton land, and significant differences were detected for any pair of groups, except for the pair of *Suaeda* land and bareland.

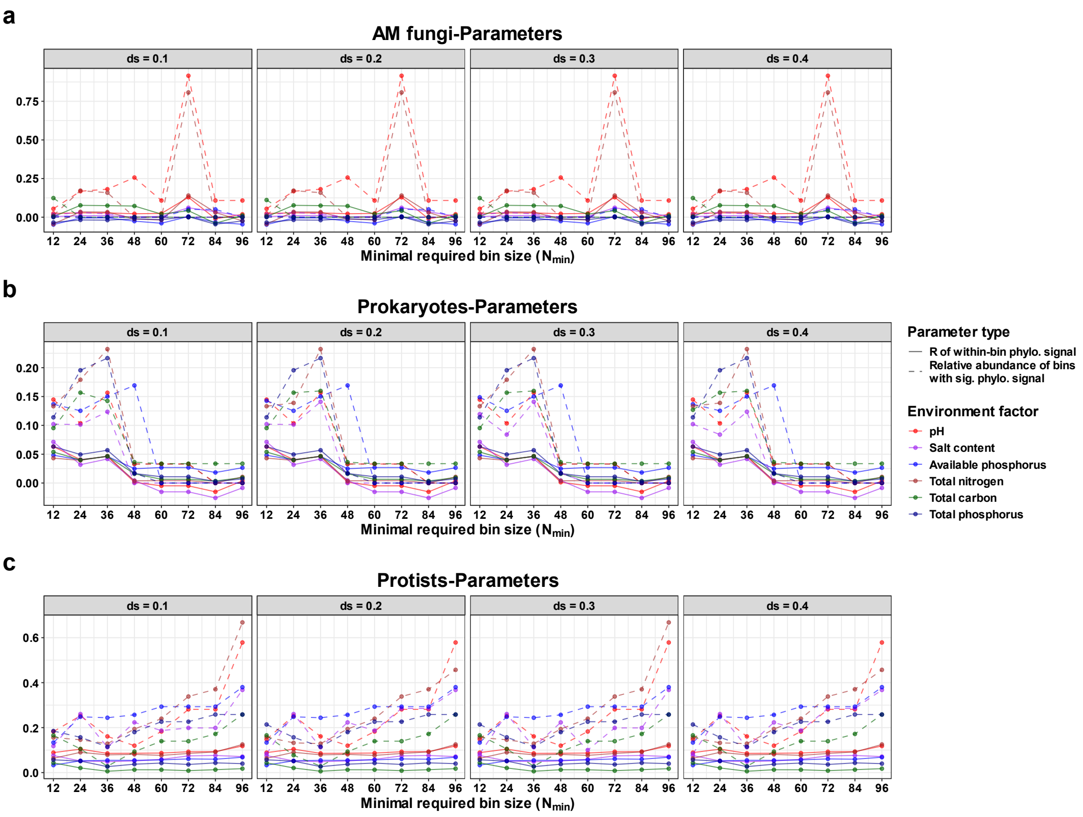

Fig. S14. Detection of optimal minimal required bin size (N_{min}) and phylogenetic signal threshold (d_s) for phylogenetic bin-based null model analysis (iCAMP). a-c Optimal N_{min} was detected with d_s from 0.1 to 0.4 using phylogenetic bin-based null model analysis (iCAMP). High relative abundance of bins with significant phylogenetic signal and average R value of all within-bin Mantel test were detected in N_{min} = 72 for **a** AM fungal community, N_{min} = 36 for **b** prokaryotic community, and N_{min} = 96 for **c** protistic community, with d_s ranging from 0.1 to 0.4.

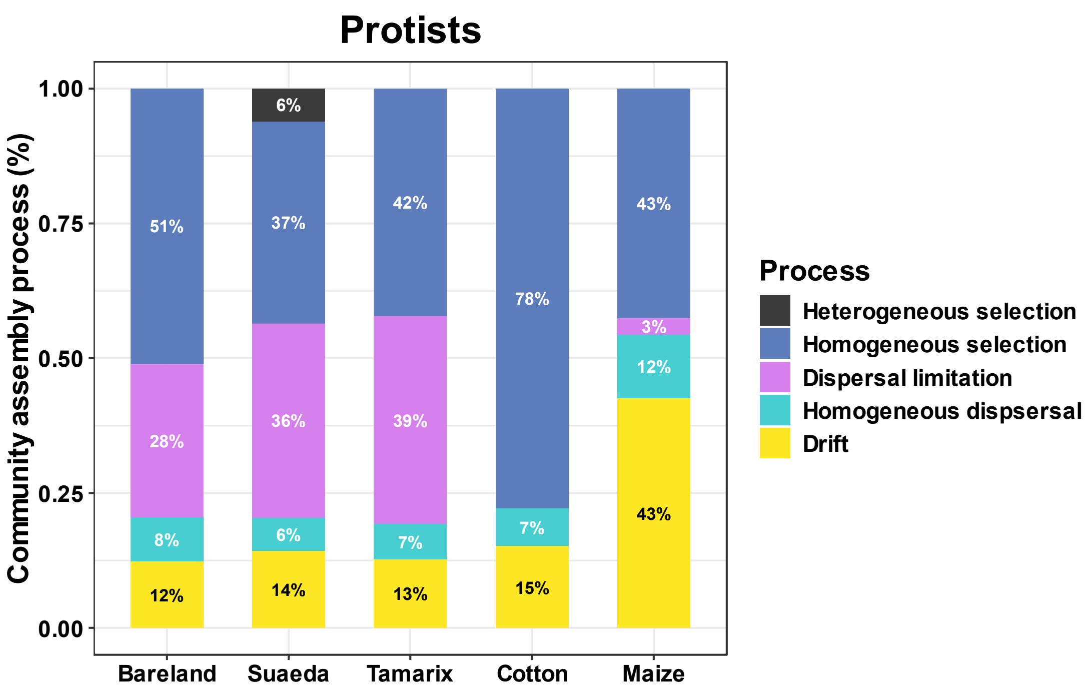

Fig. S15. Higher relative importance dispersal limitation and lower relative importance of homogeneous selection in wildland than in farmland for protistic community composition. The relative importance of dispersal limitation is higher in bareland, *Suaeda*, and *Tamarix* lands than in cotton and maize lands, whereas the relative importance of homogeneous selection is higher in cotton and maize lands than in bareland, *Suaeda,* and *Tamarix* lands. Percentages for processes with relative importance greater than 1% are displayed in each habitat.

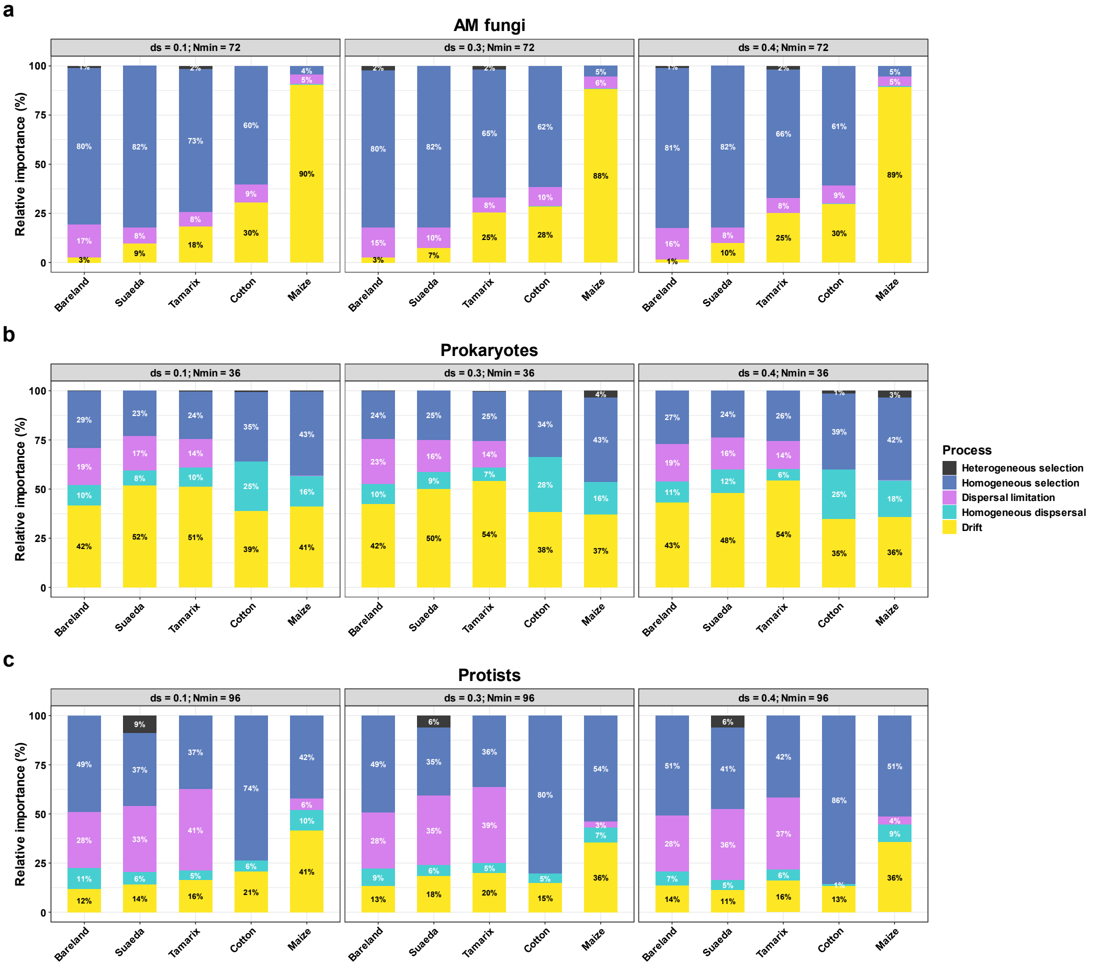

Fig. S16. Similar community assembly processes were detected using optimal N_{min} and different phylogenetic signal thresholds (d_s) for each taxon. Community assembly processes were calculated by phylogenetic bin-based null model analysis (iCAMP) using different d_s ($d_s = 0.1$, $d_s = 0.3$, $d_s = 0.4$) and optimal N_{min} for **a** AM fungal community, **b** prokaryotic community, and **c** protistic community. The patterns are similar in different d_s conditions (including $d_s = 0.2$, which was used in the main text). Percentages for processes with relative importance greater than 1% are displayed in each habitat.

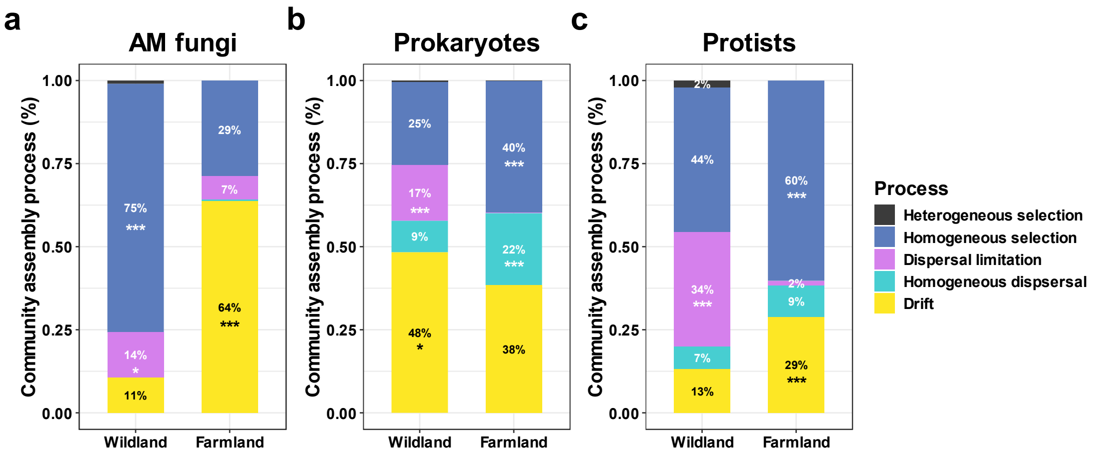

Fig. S17. Differences in community assembly processes between wildland and farmland. a For AM fungal community, homogeneous selection and dispersal limitation have significantly higher relative importance, while drift has significantly lower relative importance in wildland than in farmland. **b** For prokaryotic community, dispersal limitation and drift have significantly higher relative importance, while homogeneous selection and homogenous dispersal have significantly lower relative importance in wildland than in farmland. **c** For protistic community, dispersal limitation has significantly higher relative importance, while homogeneous selection and drift have significantly lower relative importance in wildland than in farmland. The differences in community assembly processes between wildland and farmland were tested using the Chi-square test. The detailed test results are presented in Table S6. The processes with significant differences are marked with an asterisk in the habitats where their relative importance is higher. *$P < 0.05$; **$P < 0.01$; ***$P < 0.001$

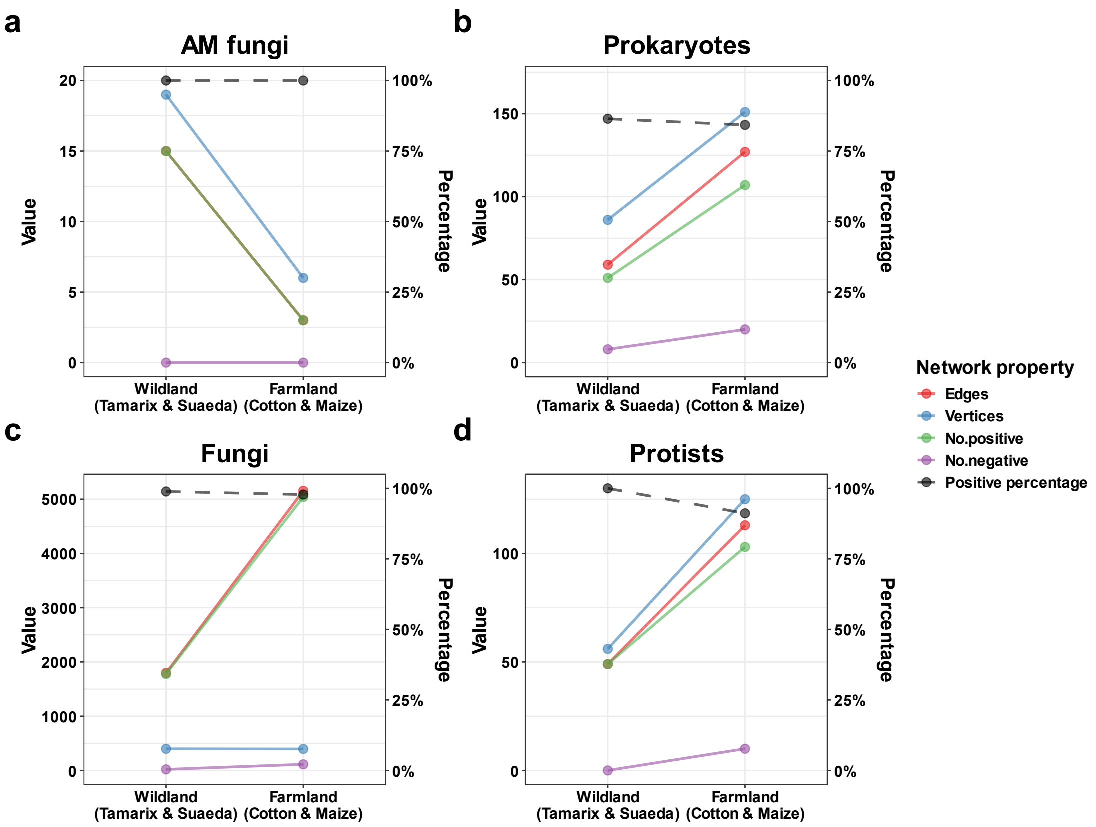

Fig. S18. Properties of co-occurrence networks of microbial community in wildland and farmland. The scatter plots show co-occurrence network properties for **a** AM fungi, **b** prokaryotes, **c** fungi, **d,** and protists in wildland and farmland.

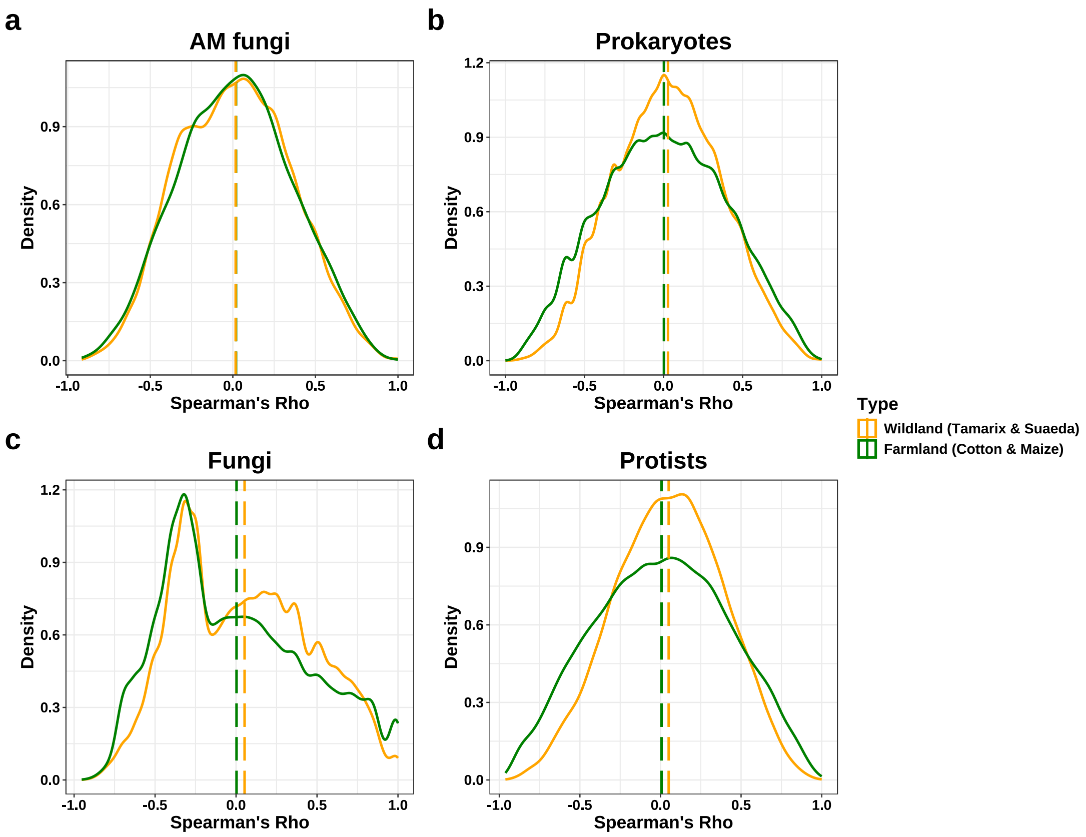

Fig. S19. Positive associations of paired OTUs are more frequent in wildland than in farmland except for AM fungi that are more frequent in farmland than in wildland. Density plot of all Spearman's Rho of paired OTUs in **a** AM fungi community, **b** prokaryotic community, **c** fungi community, and **d** protist community. The dotted lines represent the mean value of all Spearman's Rho.

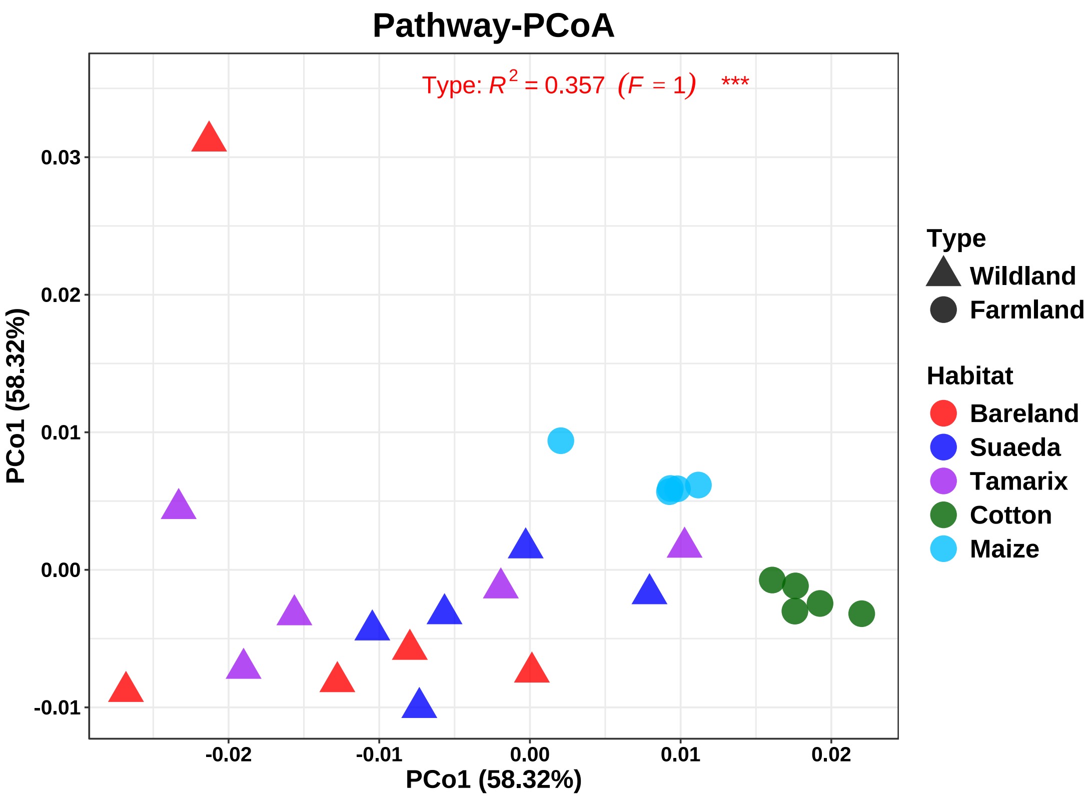

Fig. S20. Habitat significantly affects the composition of prokaryotic KOs. Bray-Curtis dissimilarity-based Principal Coordinated Analysis (PCoA) followed by Permutational Multivariate Analysis of Variance (PERMANOVA) shows habitat type explains 37.5% of the variance in KO composition.

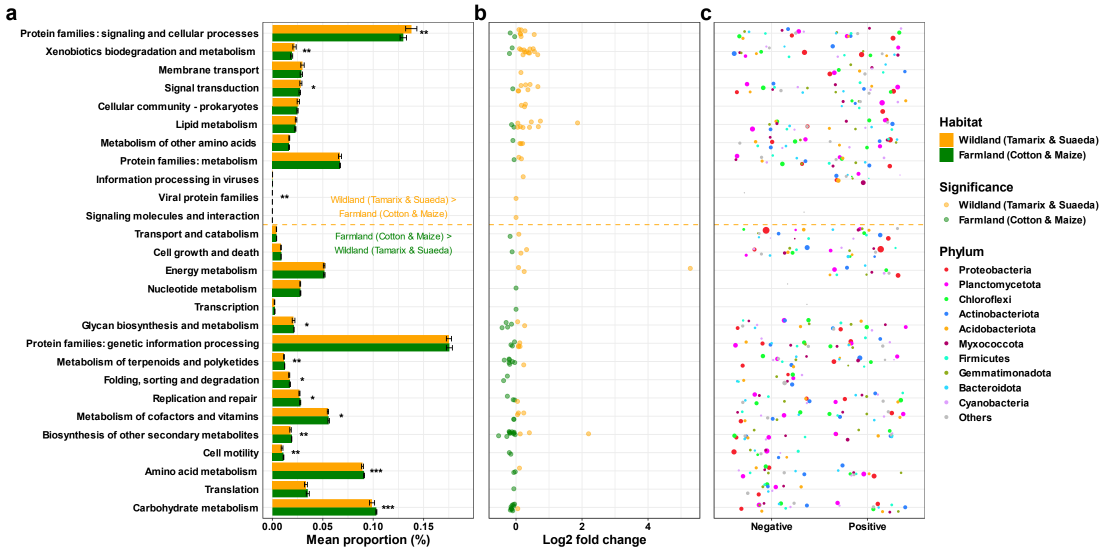

Fig. S21. Differential analysis of KOs in wildland and farmland and taxa contribution to different pathways. a Barplot depicts the mean proportion of KO categories at level 2 for prokaryotic communities in farmland (colored in green) and wildland (colored in yellow). KO categories with significant differences between farmland and wildland are labeled by asterisks, and the *P* values are adjusted by the false discovery rate (FDR) method. **b** Scatter plot shows the KOs with significantly different abundance in wildland and farmland. **c** The scatter plot shows the contribution of each prokaryotic phylum on different pathways. The differential KOs was detected using the DEseq2 method.

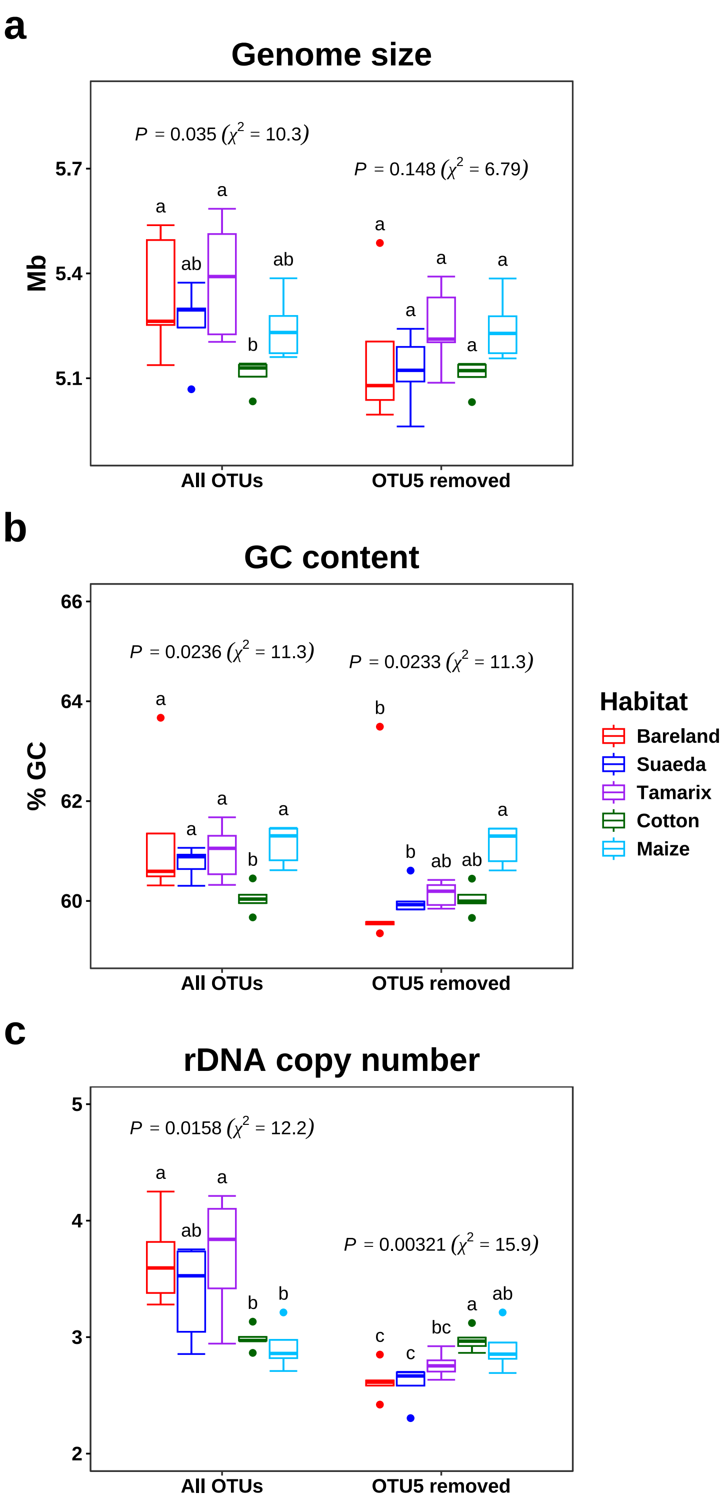

Fig. S22. Prokaryotic functional traits across five habitats. a Genome size, **b** GC content, and **c** rDNA copy number of all OTUs and all OTUs but removing OTU5 (*Acinetobacter*) in five habitats. The differences were detected by the Kruskal-Wallis

test with the *P* value adjusted by the false discovery rate (FDR) method.

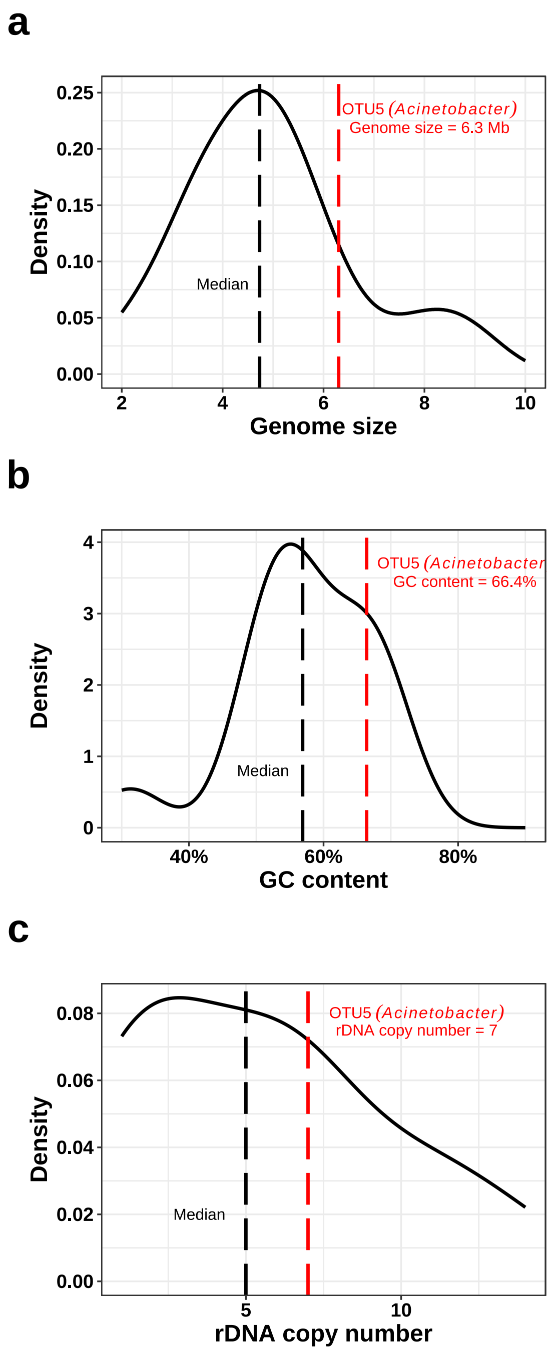

Fig. S23. Genome size, GC content, and rDNA copy number of OTU5 (*Acinetobacter*) are higher than the median values of those of all significantly differential OTUs. Density plot of **a** genome size, **b** GC content, and **c** rDNA copy number of all significantly differential prokaryotic OTUs, among which the black dot

lines represent the median value of three traits respectively, and the red dot lines represent the values of three traits of OTU5 (*Acinetobacter*).

Supplementary tables

Table S1. Primer pairs for amplicon PCR experiment

AM fungi	GeoA2/AML2 [1, 2]<br>5’-CCAGTAGTCATATGCTTGTCTC-3’/5’-GAACCCAAACACTTTGGTTTCC-3’<br>NS31/AMDGR [3, 4]<br>5’-TTGGAGGGCAAGTCTGGTGCC-3’/5’-CCCAACTATCCCTATTAATCAT-3’
Prokaryotes	515F/806R [5]<br>5’-GTGCCAGCMGCCGCGGTAA-3’/5’-GGACTACHVGGGTWTCTAAT-3’
Fungi	fITS7/ITS4F [6, 7]<br>5’-GTGARTCATCGAATCTTTG-3’/5’-AGCCTCCGCTTATTGATATGCTTAART-3’
Protist	TAReuk454FWD1/V4 18S Next.Rev [8, 9]<br>5’-CCAGCASCYGCGGTAATTCC-3’/5’-ACTTTCGTTCTTGATYRATGA-3’

Table S2. 20μl PCR solution

	Volume/μl
Sterile deionized water (Solarbio)	2.5
2 × Phanta Flash Master Mix (Dye Plus) (Vazyme, Beijing)	10
Bovine serum albumin (0.3%) (OKA, Beijing)	2.5
Forward primers (1μM)	2.5
Reverse primers (1μM)	2.5
Template DNA (5 ng μl^{-1})*	2.5

*For AM fungi, the products of the first round PCR were diluted to 5 ng μl^{-1}, and then used as template DNA for the second round PCR.

Table S3. thermal cycling conditions of PCR

	AM fungi	Bacteria	Fungi	Protist
(1) Pre-denaturation	98℃ (30 s)	98℃ (30 s)	98℃ (30 s)	98℃ (30 s)
(2) Denaturation	98℃ (10 s)	98℃ (10 s)	98℃ (10 s)	98℃ (10 s)
(3) Annealing	58℃ (30 s)	56℃ (5 s)	58℃ (5 s)	58℃ (30 s)
(4) Extension	72℃ (40 s)	72℃ (40 s)	72℃ (40 s)	72℃ (40 s)
(5) End	72℃ (1 min)	72℃ (1 min)	72℃ (1 min)	72℃ (1 min)
Cycle running (2)-(4)	20 cycles in round1<br>35 cycles in round2	35 cycles	35 cycles	35 cycles

Table S4. Soil physiochemical properties

**Sample id**	**pH**	**Salt content (‰)**	**Available phosphorus (mg/kg)**	**Total nitrogen (%)**	**Total carbon (%)**	**Total phosphorus (%)**
maize1	7.840	0.780	3.300	0.095	2.996	0.065
maize2	7.790	0.790	4.000	0.092	3.114	0.069
maize3	7.770	0.650	3.600	0.110	3.136	0.067
maize4	7.790	0.370	2.800	0.097	3.138	0.065
maize5	7.770	0.610	4.400	0.078	2.962	0.063
cotton1	7.730	0.560	12.500	0.096	2.471	0.077
cotton2	7.560	1.510	17.500	0.105	2.472	0.080
cotton3	7.630	1.260	11.300	0.096	2.513	0.077
cotton4	7.540	1.810	17.500	0.114	2.580	0.080
cotton5	7.600	0.980	10.100	0.096	2.394	0.084
*Tamarix*1	7.210	12.130	10.400	0.131	2.104	0.032
*Tamarix*2	7.320	12.650	5.200	0.077	1.495	0.024
*Tamarix*3	7.150	12.030	8.800	0.123	1.992	0.031
*Tamarix*4	7.250	10.570	5.800	0.063	1.213	0.023
*Tamarix*5	7.300	10.710	3.700	0.066	1.418	0.021
*Suaeda*1	8.720	43.080	2.900	0.043	1.596	0.029
*Suaeda*2	8.740	45.260	2.200	0.038	1.582	0.030

*Suaeda*3	8.800	63.170	3.400	0.067	1.937	0.029
*Suaeda*4	8.730	39.440	1.700	0.042	1.630	0.030
*Suaeda*5	8.850	60.560	2.100	0.040	1.597	0.026
bareland1	8.830	17.510	1.700	0.022	0.743	0.013
bareland2	8.810	18.190	1.800	0.035	0.803	0.012
bareland3	8.790	29.710	2.000	0.035	1.184	0.022
bareland4	8.790	42.800	2.200	0.044	1.457	0.025
bareland5	8.890	28.190	2.200	0.063	1.580	0.025

Table S5. Topological parameters of microbial co-occurrence network

	AM fungi		Prokaryotes		Fungi		Protists	
	Wildland	Farmland	Wildland	Farmland	Wildland	Farmland	Wildland	Farmland
**Edge**	15.00	3.00	59.00	127.00	1797.00	5154.00	49.00	113.00
**Vertice**	19.00	6.00	86.00	151.00	399.00	396.00	56.00	125.00
**Average degree**	1.58	1.00	1.37	1.68	9.01	26.03	1.75	1.81
**Average path length**	1.00	1.00	1.05	2.11	5.07	4.61	1.02	2.97
**Diameter**	1.00	1.00	2.00	7.00	17.00	13.00	2.00	8.00
**Connectance**	0.09	0.20	0.02	0.01	0.02	0.07	0.03	0.01
**Clustering coefficient**	1.00	*	0.92	0.60	0.80	0.92	0.95	0.58
**Modularity**	0.77	0.67	0.96	0.94	0.73	0.40	0.88	0.90

*Clustering coefficient of AM fungi cannot be calculated due to few connecting vertices (less than 3 vertices).

Table S6. Results from Chi-square test for community assembly process between wildland and farmland

Taxon	Process	Chi-squared	df	p-value	significance
**AM fungi**	Homogeneous selection	101.68	1	< 2.2e-16	***
	Dispersal limitation	4.6279	1	0.03146	*
	Drift	153.26	1	< 2.2e-16	***
**Prokaryotes**	Homogeneous selection	11.25	1	0.000796	***
	Homogeneous dispersal	13.555	1	0.000232	***
	Dispersal limitation	35.544	1	2.49e-09	***
	Drift	4.4022	1	0.03589	*
**Protists**	Homogeneous selection	12.689	1	0.000368	***
	Homogeneous dispersal	0.70062	1	0.4026	
	Dispersal limitation	75.509	1	< 2.2e-16	***
	Drift	18.025	1	2.18E-05	***

*$P < 0.05$; **$P < 0.01$; ***$P < 0.001$

References

[1] J. Lee, S. Lee, J.P.W. Young, Improved PCR primers for the detection and identification of arbuscular mycorrhizal fungi, FEMS Microbiol. Ecol. 65 (2008) 339-349.

[2] D. Schwarzott, A. Schüßler, A simple and reliable method for SSU rRNA gene DNA extraction, amplification, and cloning from single AM fungal spores, Mycorrhiza. 10 (2001) 203-207.

[3] K. Sato, Y. Suyama, M. Saito, et al., A new primer for discrimination of arbuscular mycorrhizal fungi with polymerase chain reaction-denature gradient gel electrophoresis, Grassl. Sci. 51 (2005).

[4] L. Simon, M. Lalonde, T. Bruns, Specific amplification of 18S fungal ribosomal genes from vesicular-arbuscular endomycorrhizal fungi colonizing roots, Appl. Environ. Microbiol. 58 (1992) 291-295.

[5] J.G. Caporaso, C.L. Lauber, W.A. Walters, et al., Global patterns of 16S rRNA diversity at a depth of millions of sequences per sample, Proc. Natl. Acad. Sci. 108 (2011) 4516-4522.

[6] K. Ihrmark, I.T. Bodeker, K. Cruz-Martinez, et al., New primers to amplify the fungal ITS2 region--evaluation by 454-sequencing of artificial and natural communities, FEMS Microbiol. Ecol. 82 (2012) 666-677.

[7] D.L. Taylor, W.A. Walters, N.J. Lennon, et al., Accurate Estimation of Fungal Diversity and Abundance through Improved Lineage-Specific Primers

Optimized for Illumina Amplicon Sequencing, Appl. Environ. Microbiol. 82 (2016) 7217-7226.

[8] D. Vaulot, S. Geisen, F. Mahe, et al., pr2-primers: An 18S rRNA primer database for protists, Mol. Ecol. Resour. 22 (2022) 168-179.

[9] R. Piredda, M.P. Tomasino, A.M. D'Erchia, et al., Diversity and temporal patterns of planktonic protist assemblages at a Mediterranean Long Term Ecological Research site, FEMS Microbiol. Ecol. 93 (2017).
